# Effects of Desert Dust and Sandstorms on Human Health: A Scoping Review

**DOI:** 10.1029/2022GH000728

**Published:** 2023-03-01

**Authors:** Kaung Suu Lwin, Aurelio Tobias, Paul Lester Chua, Lei Yuan, Ramita Thawonmas, Sophearen Ith, Zin Wai Htay, Lin Szu Yu, Lisa Yamasaki, Marta Roqué, Xavier Querol, Julia C. Fussell, Kari Christine Nadeau, Massimo Stafoggia, Najat A. Saliba, Chris Fook Sheng Ng, Masahiro Hashizume

**Affiliations:** ^1^ Department of Global Health Policy Graduate School of Medicine The University of Tokyo Tokyo Japan; ^2^ Institute of Environmental Assessment and Water Research Spanish Council for Scientific Research Barcelona Spain; ^3^ School of Tropical Medicine and Global Health Nagasaki University Nagasaki Japan; ^4^ School of Medicine Nagasaki University Nagasaki Japan; ^5^ Iberoamerican Cochrane Centre ‐ Institut d’Investigació Biomèdica Sant Pau (IIB SANT PAU) Barcelona Spain; ^6^ Centro de Investigación Biomédica en Red de Epidemiología y Salud Pública (CIBERESP) Madrid Spain; ^7^ National Institute for Health Research Health Protection Research Unit in Environmental Exposures and Health School of Public Health Imperial College London London UK; ^8^ Sean N Parker Center for Allergy & Asthma Research Stanford University Mountain View CA USA; ^9^ Department of Epidemiology Lazio Region Health Service Rome Italy; ^10^ Faculty of Arts and Sciences American University of Beirut Beirut Lebanon

**Keywords:** desert dust, scoping review, sandstorm, epidemiology, health

## Abstract

Desert dust and sandstorms are recurring environmental phenomena that are reported to produce serious health risks worldwide. This scoping review was conducted to identify the most likely health effects of desert dust and sandstorms and the methods used to characterize desert dust exposure from the existing epidemiological literature. We systematically searched PubMed/MEDLINE, Web of Science, and Scopus to identify studies that reported the effects of desert dust and sandstorms on human health. Search terms referred to desert dust or sandstorm exposure, names of major deserts, and health outcomes. Health effects were cross‐tabulated with study design variables (e.g., epidemiological design and methods to quantify dust exposure), desert dust source, health outcomes and conditions. We identified 204 studies that met the inclusion criteria for the scoping review. More than half of the studies (52.9%) used a time‐series study design. However, we found a substantial variation in the methods used to identify and quantify desert dust exposure. The binary metric of dust exposure was more frequently used than the continuous metric for all desert dust source locations. Most studies (84.8%) reported significant associations between desert dust and adverse health effects, mainly for respiratory and cardiovascular mortality and morbidity causes. Although there is a large body of evidence on the health effects of desert dust and sandstorms, the existing epidemiological studies have significant limitations related to exposure measurement and statistical analysis that potentially contribute to inconsistencies in determining the effect of desert dust on human health.

## Key Points

- Desert dust and sandstorms are recurring environmental phenomena that are reported to produce serious health risks worldwide
- Inconsistencies in exposure definitions and modeling strategies may have contributed to the observed heterogeneity in the effect estimates
- Developing a standardized research protocol could be a vital step toward better assessing the association between desert dust and health

## 1 Introduction

Desert dust and sandstorms are recurring environmental phenomena that are reported to produce serious health risks worldwide (Goudie, 2014; Shao et al., 2011). Because of the desertification caused by deforestation, climate change, and human activities, these phenomena have increased in frequency and intensity in a wide geographical area in recent years (United Nations Environment Programme, 2016). Desert dust and sandstorms play a significant role in weather, climate, and atmospheric chemistry. (De Longueville et al., 2010; Mahowald et al., 2010). Desert dust may be a serious hazard to the environment and human health, given its effect on the air quality, whether nearby or thousands of kilometers from deserts or dust emission sources (Ginoux et al., 2012; Prospero et al., 2002). Depending on weather and climate, desert dust can remain suspended in the atmosphere for days, causing allergy outbreaks far from its source (Mori et al., 2003; Rodríguez et al., 2011).

The physical, biological, and chemical properties of dust particles can potentially cause detrimental effects on human health (Mori et al., 2003; Zhang et al., 2016). Desert dust affects air quality by increasing ambient particulate matter (PM) concentrations by carrying large loads of mineral or crustal dust and anthropogenic pollutants that accumulate locally in source areas or are trapped in high dust air masses (Mori et al., 2003; Rodríguez et al., 2011). Significant loads of microorganisms and toxic biogenic allergens may be transported via these processes (Griffin et al., 2001; Ho et al., 2005).

Previous studies have observed evidence of associations between exposure to desert and sandstorm dust and morbidity or mortality (Griffin et al., 2001; Ho et al., 2005; Rodríguez et al., 2011; Zhang et al., 2016). Some studies have suggested that dust particle size is one of the key factors affecting potential health risks (Griffin et al., 2001; Zhang et al., 2016). Large‐sized dust particles can potentially damage external organs, causing skin, eye, and ear irritation, whereas small‐sized dust particles can enter the respiratory tract and cause respiratory disorders (Griffin et al., 2001; Ho et al., 2005; Rodríguez et al., 2011; Zhang et al., 2016). Small particles may also penetrate the respiratory tract and damage the cardiovascular, cerebral, cerebrovascular, blood, and immune systems. Furthermore, infectious diseases such as meningococcal meningitis and Rift Valley fever can be spread by desert dust (Griffin et al., 2001; Ho et al., 2005; Rodríguez et al., 2011; Zhang et al., 2016).

Despite findings from previous epidemiological studies that have suggested the varied potential health effects (i.e., adverse effect, no effect, and protective effect) of desert dust, the evidence remains inconclusive, and results are inconsistent (Griffin et al., 2001; Ho et al., 2005; Rodríguez et al., 2011; Zhang et al., 2016). There are some existing reviews, systematic or not, on the health effect of desert dust, but these studies are limited to specific geographical areas and health outcomes. (Hashizume M et al., 2020; Domínguez‐Rodríguez A et al., 2021). For example, skin symptoms and allergic reactions have not been considered (Otani et al., 2012). Moreover, most of these reviews have not defined an appropriate research question based on a well‐defined population, exposure, comparator, and outcomes (i.e., PECOs). Therefore, inconsistent results have been reported across studies and geographic regions. The main sources of heterogeneity across studies were methodological and related to study design, exposure assessment methods for identifying dust episodes and quantifying the dust and non‐dust PM contributions, and the exposure metric to investigate health effects (Querol et al., 2019; Tobías & Stafoggia, 2020). In addition, the health effects in dust‐emitting hotspots (i.e., emission areas within proximity to the source of desert dust) may differ from those occurring in distant dust receptor areas (i.e., locations far from the source of desert dust) where dust is diluted and mixed with other pollutants (Querol et al., 2019). These limitations and challenges have prevented the quantitative summary of evidence through meta‐analysis and the assessment of evidence quality (e.g., by applying the GRADE system) (Guyatt et al., 2008). As a result, existing reviews may provide a limited view of the impact of desert dust and sandstorms on human health.

Methodological studies incorporating exposure and outcome assessment are needed to pave the way for more rigorous systematic reviews that can provide an enhanced understanding of the impact of desert dust and sandstorms on human health. This scoping review aims to identify and classify the methods used to characterize desert dust exposure and the most likely health effects of desert dust and sandstorms from the existing epidemiological literature. The primary research question for this scoping review followed the Population–Concept–Context mnemonic by including the methodological characteristics of the epidemiological studies (i.e., concept) that assessed the health effects of desert dust and sandstorms in populations living in dust‐emitting hotspots and receptor areas (i.e., population and context). The following research sub‐areas focus on specific study characteristics in the existing scientific literature: (a) health outcomes associated with desert dust exposure, (b) methods used to identify and quantify desert dust exposure and non‐desert dust PM components in health studies conducted during dust outbreaks, (c) susceptible populations and affected areas, and (d) the type of epidemiological study designed to identify the association between dust exposure and health outcomes.

The evidence generated from this scoping review may facilitate the identification and selection of subject areas for further review and evaluation by the World Health Organization Global Air Pollution and Health‐Technical Advisory Group (WHO‐GAPH‐TAG) on Desert Dust and Health and identify knowledge gaps for future research.

## 2 Materials and Methods

This scoping review followed the guidelines of the Joanna Briggs Institute (Peters et al., 2015). It was conducted following a developed protocol (Hashizume et al., 2022) in accordance with the Preferred Reporting Items for Systematic Reviews and Meta‐Analyses for Scoping Review (PRISMA‐ScR) reporting guidelines to ensure methodological reliability (Tricco et al., 2018).

### 2.1 Search Strategy

The literature search was performed using the online databases of PubMed/MEDLINE, Web of Science, and Scopus. The search terms used referred to desert dust or sandstorm exposure (e.g., dust episodes or events, calima, Kosa, and yellow dust), names of major deserts (e.g., Sahara and Gobi), and general health outcomes. The full search syntax used for each database can be found in Appendix A in Supporting Information S1).

### 2.2 Eligibility Criteria

The scoping review included English language epidemiological research articles published in peer‐reviewed journals until 31 December 2021. We included primary research using any observational study design, including time‐series, case‐crossover, longitudinal, cohort, cross‐sectional, case‐control, and case report. Experimental studies were only considered if human subjects were involved (i.e., controlled human exposure studies). Health impact assessment studies using the exposure‐risk function derived from the previous epidemiological evidence were excluded since the results from these studies are not comparable with those from epidemiological studies. All types of literature reviews, including but not limited to narrative, scoping, and systematic reviews, were excluded. We further excluded conference abstracts, letters, and editorials unless they reported the results of epidemiological analysis.

#### 2.2.1 Participants

We included all studies conducted on humans irrespective of age, sex, health status, and geographic location. Studies in animal models and cell culture or in vitro studies were excluded.

#### 2.2.2 Exposure

This scoping review considered exposure to mineral dust or anthropogenic PM and mixed PM resulting from desert and sandstorm episodes and characterized using any methodology or metric (Querol et al., 2019; Tobías & Stafoggia, 2020). Studies that only investigated the effect of PM unrelated to desert dust or sandstorm were excluded.

#### 2.2.3 Outcomes

This scoping review included all health outcomes, including mortality, hospital admissions or visits, ambulance transport, emergency room visits, clinically diagnosed or self‐reported symptoms, and human body system dysfunction. We also extracted information on health conditions, including respiratory diseases, cardiovascular diseases, cerebrovascular diseases, allergic skin and eye problems, infectious diseases, cancer and metabolic disease, adverse birth outcomes, accidents and injuries, maternity and reproduction, under‐5 mortality, mental health, health‐related quality of life and school absence due to sickness.

#### 2.2.4 Context

All original epidemiological research studies that directly describe the health effects of desert dust and sandstorms were considered in this scoping review. Health effects described in dust‐emitting hotspots and receptor areas were included.

### 2.3 Study Selection, Data Extraction, and Summary of Results

All records were imported and deduplicated in EndNote X9 (Clarivate Analytics) following conventional guidelines (Bramer et al., 2016). At least two reviewers independently screened titles and abstracts. All studies were classified for inclusion or exclusion based on the eligibility criteria and further checked for duplication. Full‐text articles were retrieved if the study's inclusion status was unclear from the title and abstract, and relevant studies were further confirmed for inclusion through full‐text review. Any discrepancy between the assessments of the two reviewers was addressed by discussion or input from a third reviewer. The screening was performed using Rayyan, a web and mobile app for systematic and scoping reviews (Ouzzani et al., 2016). A PRISMA flow diagram was generated after all articles to be included were identified.

A data extraction form in an Excel spreadsheet (Microsoft Corporation) was used to guide data collection from selected studies and to map the evidence. Reviewers extracted the following data for each study.

1. Citation details (e.g., first author, title, journal, and publication date).
2. Study area (e.g., city, country, and emission or receptor region) and period.
3. Characteristics of the study population (e.g., age, sex.)
4. Study design.
5. Methods to identify dust episodes or quantify dust contribution to PM.
6. Health outcomes (e.g., mortality, hospital admissions or visits, ambulance transport), and health conditions (e.g., respiratory diseases, cardiovascular diseases, cerebrovascular diseases) assessed.
7. Health effects (i.e., adverse, non‐significant, and protective) reported.

Descriptive tables were developed to summarize the type of primary studies included. Results were classified and organized by desert dust source, dust exposure assessment method, health outcomes, and health condition. Additionally, health effects were cross‐tabulated with study design variables (i.e., epidemiological designs, methods to quantify dust exposure), desert dust source, health outcomes, and health condition studied.

### 2.4 Quality Assessment

In line with scoping review guidelines, study quality or formal risk of bias was not assessed nor used as a basis for study exclusion (Peters et al., 2015; Tricco et al., 2018).

### 2.5 Ethical Approval

Not applicable given this scoping review was limited to descriptive narrative analysis of published studies. This scoping review protocol is available at https://osf.io/vqsje.

## 3 Results

**Figure 1:**
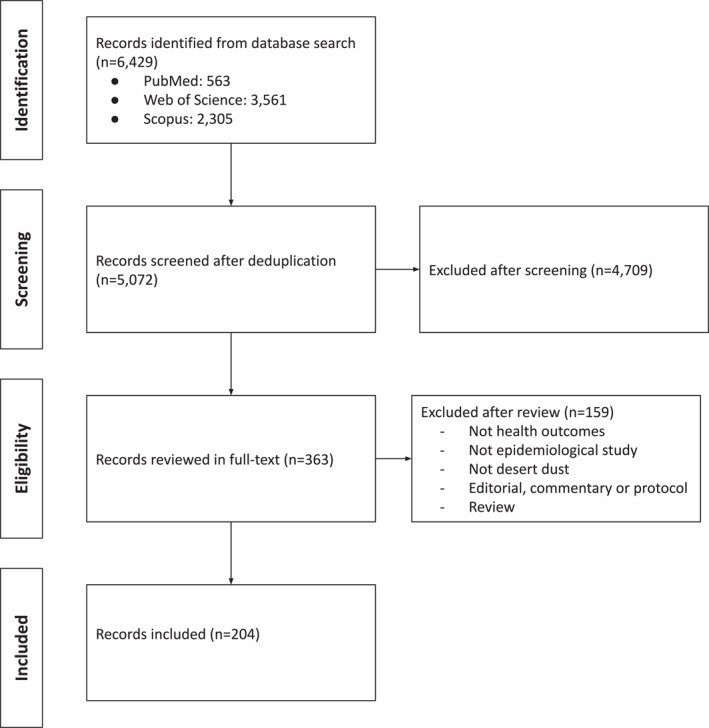
Flow chart of selected studies.

After deduplication, the searches in PubMed/MEDLINE, Web of Science, and Scopus databases identified a total of 5,072 studies (Figure 1), of which 4,709 studies were excluded after reviewing the title and abstract. Among the 363 studies remaining for full‐text review, 204 met the inclusion criteria (Figure 1).

**Figure 2:**
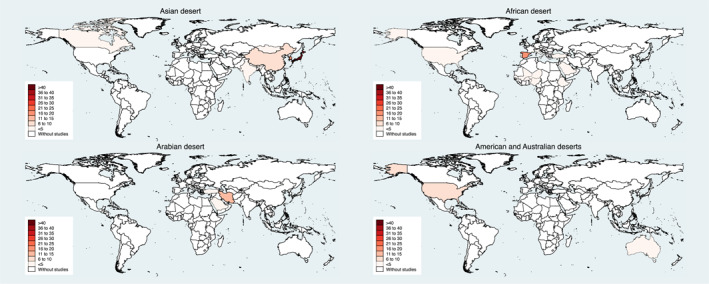
Geographical areas where studies for health effects of desert dust have been conducted. Note: The higher the color intensity, the more studies for health effects of respective desert dust have been conducted in that area.

Among the 204 studies included in the review (Supplementary Table 1 in Supporting Information S1), 107 (52.2%) investigated the health effects of Asian desert dust. The remaining 58 (28.3%), 26 (12.7%), 10 (4.9%), and 4 (2.0%) studies analyzed the health effects of African, Arabian, American, and Australian desert dust, respectively, and one study involved both African and American desert dust (Table 1). The Asian desert dust studies were conducted mainly in Japan, Taiwan, and South Korea. Most studies investigating the health effects of African desert dust were conducted in Spain, while most Arabian desert dust studies were from Iran (Supplementary Table 2 in Supporting Information S1). Figure 2 shows the geographical areas where studies were conducted. The highest number of studies were published in 2014 (Supplementary Figure 1 in Supporting Information S1). Asian deserts (e.g., the cold desert areas of Gobi and Taklamakan) are the main source of Asian desert dust, which can be transported to Canada in North America. Similarly, African deserts (e.g., the hot desert areas of the Sahara and the Sahel) are the main source of African desert dust, which can also reach the United States and Mexico. Arabian (e.g., Arabian Peninsula), American (e.g., Mojave), and Australian (e.g., Great Sandy) deserts are the main sources of desert dust in the Arabian Peninsula, Australia, and America, respectively (Supplementary Figure 2 in Supporting Information S1).

**Table 1:** Descriptive Characteristics of Selected Studies (*N* = 204)

Characteristic	Number of studies	(%)
Desert dust source^a^		
Asian	107	(52.2)
African	58	(28.3)
Arabian	26	(12.7)
American	10	(4.9)
Australian	4	(2.0)
Study design		
Time‐series	108	(52.9)
Case‐crossover	40	(19.6)
Longitudinal or Cohort	29	(14.2)
Cross‐sectional or Case‐control	24	(11.8)
Other^b^	3	(1.5)
Health effect		
Adverse	173	(84.8)
Non‐significant	29	(14.2)
Protective	1	(0.5)
Not applicable	1	(0.5)

^a^ One study examined both desert dust exposure from African and American sources.

^b^ Others include case reports (*n* = 1), experimental (*n* = 1), and quasi‐experimental (*n* = 1) study designs.

More than half of the studies, 52.9%, used a time‐series design, whereas 19.6% employed a case‐crossover, 14.2% a longitudinal or cohort, and 11.8% a cross‐sectional or case‐control design (Table 1). Most studies (84.8%) reported a significant association between desert dust and adverse health effects (Table 1).

### 3.1 Measurement of Dust Exposure by Desert Dust Source

Desert dust exposure was measured using a multi‐tool approach, which is a combination of methods, including direct measurement of surface dust concentration as PM10 or PM2.5 (i.e., containing desert dust and other PM components), ensemble models, satellite retrievals, and back trajectories in 34.6% of Asian and 53.4% of African desert dust studies (Table 2). The threshold value method based on PM exceedances was used in 38.5% of Arabian and 50.0% of Australian desert studies. As measured by visibility, dust exposure was found in 16.8%, 11.5%, and 3.4% of studies of Asian, Arabian, and African deserts, respectively. The remaining studies measured exposure using source appointment (including chemical composition), aerosol in remote sensing (aerosol optical depth), active remote sensing (light detection and ranging), and modeling (including forecast model, satellite, or reanalysis data for desert dust). Twenty‐two (10.7%) studies did not provide details of dust exposure measurement.

**Table 2:** Methods for Desert Dust Exposure Measurement by Desert Dust Source

Dust exposure method		Asian (*N* = 107)		African (*N* = 58)		Arabian (*N* = 26)			American (*N* = 10)				Australian (*N* = 4)	
		*n*	(%)	*n*	(%)	*n*	(%)		*n*		(%)		*n*	(%)
Multi‐tool approach (*N* = 73)		37	(34.6)	31	(53.4)	3	(11.5)		2		(20.0)		0	(0.0)
Threshold value (*N* = 41)		19	(17.8)	8	(13.8)	10	(38.5)		2		(20.0)		2	(50.0)
Remote sensing (*N* = 27)		17	(15.9)	7	(12.1)	2	(7.7)		1		(10.0)		0	(0.0)
Visibility (*N* = 23)		18	(16.8)	2	(3.4)	3	(11.5)		0		(0.0)		0	(0.0)
Modeling (*N* = 13)		5	(4.7)	4	(6.9)	1	(3.8)		3		(30.0)		0	(0.0)
Source appointment (*N* = 6)		2	(1.9)	2	(3.4)	1	(3.8)		0		(0.0)		1	(25.0)
Not reported (*N* = 22)		9	(8.4)	4	(6.9)	6	(23.1)		2		(20.0)		1	(25.0)

*Note.* Multi‐tool approach (combination of methods including ensemble models, satellite retrievals and back trajectories); threshold value (based on PM exceedances); remote sensing (includes AOD and LIDAR); visibility (includes reporting dust events/storms from other sources); modeling (includes any type of forecast model, satellite or reanalysis data for desert dust); source apportionment (includes chemical composition); not reported (or not properly described).

### 3.2 Dust Exposure Metric by Desert Dust Source

Most of the studies in Asian (69.4%) and Arabian (61.5%) deserts assessed the effect of dust exposure using a binary risk factor, examining if the occurrence of a given health outcome was higher on dust days than on non‐dust days (Table 3). The binary metric as risk exposure was less used in African desert studies (35.9%). Moreover, the binary metric was also used as an effect modifier of the association between the health outcome and daily concentrations of PM air pollution between dust days and non‐dust days, was mainly used in African desert dust studies (31.2%) and to a lesser extent in Asian (15.7%) and Arabian (11.5%) desert studies. Very few used the binary metric as a confounder when examining if the association between a health outcome and daily PM concentrations was independent of dust events. Dust exposure using a continuous metric has mainly been used in Asian and African desert studies to examine if desert and non‐desert dust sources of PM air pollution are independently associated with a health outcome (5.6% and 4.7%, respectively). However, only in two African desert studies (6.3%) the continuous metric has been extended to examine if the association is different between dust days and non‐dust days and whether these associations are independent. Other studies in Asian (1.9%) and African (7.2%) deserts also examined the association between health outcomes and the average increase of desert dust. Many American desert studies (80.0%) and all Australian studies (100%) used the binary metric as a risk factor.

**Table 3:** Dust Exposure Metric by Desert Dust Source

		Asian (*N* = 108)		African (*N* = 64)		Arabian (*N* = 26)			American (*N* = 10)		Australian (*N* = 4)	
Dust exposure metric^a^		*n*	(%)	*n*	(%)	*n*	(%)		*n*	(%)	*n*	(%)
Binary												
Risk factor^b^ (*N* = 126)		75	(69.4)	23	(35.9)	16	(61.5)		8	(80.0)	4	(100.0)
Confounder^c^ (*N* = 5)		1	(0.9)	1	(1.6)	2	(7.7)		1	(10.0)	0	(0.0)
Effect modifier^d^ (*N* = 40)		17	(15.7)	20	(31.2)	3	(11.5)		0	(0.0)	0	(0.0)
Continuous												
Risk factor^e^ (*N* = 7)		2	(1.9)	4	(6.3)	1	(3.8)		0	(0.0)	0	(0.0)
Two sources^f^ (*N* = 9)		6	(5.6)	3	(4.7)	0	(0.0)		0	(0.0)	0	(0.0)
Three sources^g^ (*N* = 2)		0	(0.0)	2	(3.1)	0	(0.0)		0	(0.0)	0	(0.0)
Not applicable (*N* = 23)		7	(6.5)	11	(17.2)	4	(15.4)		1	(10.0)	0	(0.0)

^a^ Categories are not mutually exclusive.

^b^ Examines if the occurrence of a health outcome is higher on dust days than in non‐dust days.

^c^ Examines if the association between a health outcome and daily concentrations of PM air pollution are independent of dust events.

^d^ Examines if the association between a health outcome and daily concentrations of PM air pollution is different on dust days and non‐dust days.

^e^ Examines the association between a health outcome and the average increase of desert dust.

^f^ Examines if desert and non‐desert dust sources of PM air pollution are independently associated with a health outcome.

^g^ Examines if the association between a health outcome and non‐desert PM air pollution is different between dust days and non‐dust days and whether these associations are independent of desert particulate matter.

Desert dust exposure metrics used to study health outcomes and conditions, described in the next section, are summarized in Supplementary Tables 3 and 4 in Supporting Information S1, respectively, while the health effects reported through the desert dust exposure metric used are summarized in Supplementary Table 5 in Supporting Information S1.

### 3.3 Health Outcomes Reported by Desert Dust Source

Studies on Asian desert dust most frequently assessed health symptoms and dysfunction (41.4%), followed by hospital admissions or visits (35.5%) and mortality (15.0%) (Table 4). These health outcomes were further commonly studied for other desert locations. In African desert dust studies, mortality (33.9%) and hospital admissions or visits (33.9%) were the most frequent outcomes, followed by symptoms and dysfunction (24.2%). In Arabian desert dust studies, hospital admissions or visits were the most common outcome (38.7%), followed by mortality (25.8%) and symptoms and dysfunctions (22.6%). The pattern is less obvious in American and Australian desert dust studies because of the small number of studies.

**Table 4:** Health Outcomes Reported by Desert Dust Source

		Asian (*N* = 107)		African (*N* = 62)		Arabian (*N* = 31)		American (*N* = 10)		Australian (*N* = 5)	
Health outcomes^a^		*n*	(%)	*n*	(%)	*n*	(%)	*n*	(%)	*n*	(%)
Hospital admissions or visits (*N* = 77)		38	(35.5)	21	(33.9)	12	(38.7)	4	(40.0)	2	(40.0)
Symptoms and dysfunction (*N* = 71)		44	(41.1)	15	(24.2)	7	(22.6)	4	(40.0)	1	(20.0)
Mortality (*N* = 48)		16	(15.0)	21	(33.9)	8	(25.8)	2	(20.0)	1	(20.0)
Emergency room visits (*N* = 14)		5	(4.7)	5	(8.1)	3	(9.7)	0	(0.0)	1	(20.0)
Ambulance transports (*N* = 5)		4	(3.7)	0	(0.0)	1	(3.2)	0	(0.0)	0	(0.0)

^a^ Categories are not mutually exclusive.

### 3.4 Health Conditions Reported by Desert Dust Source

Respiratory diseases were the most frequently studied health condition in all source locations, ranging from 30.8% to 44.9% (Table 5). Moreover, cardiovascular (19.6%–25.0%) and all‐cause (9.5%–15.9%) diseases were the second and third most frequently studied conditions, respectively. Other less studied health conditions included infectious diseases, allergic skin and eye problems, cerebrovascular, adverse birth outcomes, allergic diseases, health‐related quality of life, under‐five mortality, accidents and injuries, maternity and reproduction, mental health, diabetes, and school absence due to sickness.

**Table 5:** Health Conditions Characteristics by Desert Dust Source

		Asian (*N* = 158)		African (*N* = 88)				Arabian (*N* = 35)		American (*N* = 13)			Australian (*N* = 8)	
Health condition^a^		*n*	(%)	*n*		(%)		*n*	(%)	*n*		(%)	*n*	(%)
Respiratory (*N* = 121)		71	(44.9)	31		(35.2)		12	(34.3)	4		(30.8)	3	(37.5)
Cardiovascular (*N* = 65)		31	(19.6)	22		(25.0)		8	(22.9)	2		(15.4)	2	(25.0)
All causes (*N* = 38)		15	(9.5)	14		(15.9)		4	(11.4)	3		(23.1)	3	(37.5)
Infectious diseases (*N* = 18)		5	(3.2)	8		(9.1)		3	(8.6)	2		(15.4)	0	(0.0)
Allergic skin and eye problems (*N* = 17)		15	(9.5)	2		(2.3)		0	(0.0)	0		(0.0)	0	(0.0)
Cerebrovascular (*N* = 10)		5	(3.2)	4		(4.5)		1	(2.9)	0		(0.0)	0	(0.0)
Adverse birth outcomes (*N* = 7)		1	(0.6)	3		(3.4)		2	(5.7)	1		(7.7)	0	(0.0)
Allergic diseases (*N* = 5)		4	(2.5)	0		(0.0)		1	(2.9)	0		(0.0)	0	(0.0)
Health related quality of life (*N* = 4)		4	(2.5)	0		(0.0)		0	(0.0)	0		(0.0)	0	(0.0)
Under‐5 mortality (*N* = 3)		1	(0.6)	3		(3.4)		0	(0.0)	0		(0.0)	0	(0.0)
Accidents and injuries (*N* = 3)		0	(0.0)	0		(0.0)		2	(5.7)	1		(7.7)	0	(0.0)
Maternity and reproduction (*N* = 3)		1	(0.6)	1		(1.1)		1	(2.9)	0		(0.0)	0	(0.0)
Mental health (*N* = 2)		2	(1.3)	0		(0.0)		0	(0.0)	0		(0.0)	0	(0.0)
Diabetes (*N* = 1)		1	(0.6)	0		(0.0)		0	(0.0)	0		(0.0)	0	(0.0)
School absence due to sickness (*N* = 1)		1	(0.6)	0		(0.0)		0	(0.0)	0		(0.0)	0	(0.0)
Not specified (*N* = 2)		1	(0.6)	0		(0.0)		1	(2.9)	0		(0.0)	0	(0.0)

^a^ Categories are not mutually exclusive.

Health conditions classified by health outcomes are summarized in Supplementary Table 6 in Supporting Information S1.

### 3.5 Health Effects Reported by Desert Dust Source

Adverse health effects were mostly reported in all desert dust source locations, ranging from 75.0% to 87.9% (Table 6). On the other hand, between 11.5% and 25.0% of the studies in their respective source locations reported no significant health effects. Only one study from the Arabian desert reported a protective health effect.

**Table 6:** Health Effects Reported by Desert Dust Source

		Asian (*N* = 107)		African (*N* = 58)			Arabian (*N* = 26)		American (*N* = 10)			Australia (*N* = 4)		
Health effect		*n*	(%)	*n*	(%)		*n*	(%)	*n*	(%)		*n*		(%)
Adverse (*N* = 174)		90	(84.1)	51	(87.9)		22	(84.6)	8	(80.0)		3		(75.0)
Non‐significant (*N* = 29)		16	(15.0)	7	(12.1)		3	(11.5)	2	(20.0)		1		(25.0)
Protective (*N* = 1)		0	(0.0)	0	(0.0)		1	(3.8)	0	(0.0)		0		(0.0)
Not applicable (*N* = 1)		1	(0.9)	0	(0.0)		0	(0.0)	0	(0.0)		0		(0.0)

*Note.* The higher the color intensity, the more studies for health effects of respective desert dust have been conducted in that area.

The health effects reported through the desert dust exposure metric are summarized in Supplementary Table 5 in Supporting Information S1, while the health effects according to the outcomes and conditions studied are summarized in Supplementary Tables 7 and 8 in Supporting Information S1, respectively.

## 4 Discussion

This scoping review highlighted which are the main health outcomes and conditions associated with desert dust exposure, and the susceptible populations and affected areas. It also provides insight of the methods used to identify and quantify desert dust exposure, and the types of epidemiological study design used to evaluate the health effects of desert dust and sandstorms.

### 4.1 Health Outcomes Associated With Desert Dust Exposure

Most of the epidemiological studies reviewed reported adverse health effects in various populations affected by differing sources of desert dust. Notably, only one study suggested a protective effect (i.e., reduced adverse health outcomes). Lee et al. (2014) observed significant adverse effects of an Asian dust storm (ADS) on mortality in Kitakyushu, Japan, and Seoul, South Korea. However, a significant negative association between ADS and mortality was observed in Taipei, Taiwan, between 2002 and 2006 (Lee et al., 2014). The authors explained that ADS has been affected by Taipei more frequently than Kitakyushu and Seoul and that Taipei's population was consequently more cautious about dust storm events (Lee et al., 2014). In contrast, Chan and Ng (2011) reported significant positive associations between ADS and mortality in Taipei between 1994 and 2007. Both studies obtained PM10 concentration measurements for ADS and non‐ADS days from the Taiwan Environmental Protection Administration. The PM10 concentration levels in Lee et al. (2014) were lower during ADS days and higher during non‐ADS days than the corresponding PM10 levels in Chan and Ng (2011). Another possible reason for the inconsistency is the difference in statistical methods employed by these two studies. A standardized dust exposure measurement and assessment protocol may therefore be beneficial in improving the consistency of findings.

This scoping review further indicates that most studies have investigated the short‐term health effects occurring during or immediately after a dust storm. The short‐term health effects of desert dust have been reported for all‐cause mortality, ambulance transports, emergency room visits, hospital admissions or visits, daily symptoms (i.e., decreased respiratory function), and other acute health conditions (Kim et al., 2012; Ko et al., 2016; Kojima et al., 2017). Only a few studies have reported long‐term health effects, including adverse birth outcomes, under‐5 mortality, and mental health (Altindag et al., 2017; Li et al., 2018; Lien et al., 2019) that occurred over a prolonged period of exposure to several episodes of a dust storm.

The types of health conditions examined in the epidemiological studies also varied by desert origin, suggesting a difference in either data availability by country/region or priorities about disease burden and vulnerability. However, the existing literature regarding the long‐term health effects of desert dust is very limited and should benefit from more research in the future.

### 4.2 Methods Used to Identify and Quantify Desert Dust Exposure

We found a substantial variation in the methods used to identify and quantify desert dust exposure. Many air quality and meteorological agencies are gradually advancing their exposure measurement methods from single measurement tools to multi‐tool approaches by combining ensemble models, satellite retrievals, and back trajectories. Therefore, a standardized protocol to identify and quantify dust exposure could help reduce the number of inconsistent findings that we observed across studies. Given the increasing availability of information related to dust storms from ground‐based observations, modeling, and remote sensing, we recommend greater collaboration between medical and environmental scientists to explore factors such as dust size fraction and specific chemical compositions harmful to human health. This cooperation could provide valuable evidence for decision‐making and implementing specific public health measures.

### 4.3 Susceptible Populations and Affected Areas

The results of the scoping review indicate that the health effects of Asian dust have been widely investigated in countries and areas in Asia, including Japan, South Korea, Taiwan, mainland China, and Hong Kong. Only one study, which assessed the impact of the 1998 Gobi dust event (an ADS) on hospital admissions in British Columbia, Canada, investigated Asian dust outside of the continent; study results indicated no significant associations (Bennett et al., 2006). However, studies investigating the health effects of African desert dust have been conducted mostly in European and other non‐African countries. Reasons may include the limited awareness of the health impact of dust, the need for health or meteorological data with sufficient accuracy and resolution, and differences in priorities in African countries. Therefore, a clear research gap exists in understanding the health effects of African desert dust on the African population.

### 4.4 Type of Epidemiological Study Designs

There are some existing reviews, systematic or not, on the health effect of desert dust, but these studies are limited to specific health outcomes and geographical areas (Domínguez‐Rodríguez et al., 2021; Hashizume et al., 2020). Evidence from this scoping review indicates that the inconsistent health effect findings are possibly due to differences in study design, exposure measurement methods, statistical methodologies, differing populations, and wide‐ranging geographical locations. Therefore, formalizing a standardized analysis protocol to quantify dust exposure and assess the health effects of desert dust is recommended. Such a protocol would guide the design and conduct of future studies, ensuring comparability among them and providing insights to shape future policy.

### 4.5 Limitations

This scoping review has some limitations. First, the search strategy was limited to original articles from peer‐reviewed journals, which may have excluded unpublished work or reports. Second, around 13% of the selected studies mentioned that dust exposure measurements were defined by the local air quality or meteorological agencies without further explanation. Although we attempted to confirm exposure measurement details by searching corresponding official websites, minimal misclassifications were possible, given that measurement methods change over time as technology improves.

## 5 Conclusions

There is considerable evidence of the health effects of desert dust and sand storms, especially for respiratory and cardiovascular mortality and morbidity causes. However, the existing epidemiological studies have significant limitations regarding exposure measurement and statistical analysis contributing to inconsistencies in determining the health impacts of desert dust. Developing a standardized research protocol defining a consistent research structure, particularly regarding exposure measurement and exposure‐outcome modeling, could be essential for better assessment of the associations between desert dust and health in forthcoming epidemiological studies. Additionally, more evidence on the health effects of African desert dust on African populations is needed. Investment in improving exposure measurement should be encouraged, and public awareness to prevent the adverse effects of desert dust should be promoted.

## Conflict of Interest

The authors declare no conflicts of interest relevant to this study.

## Supporting information

Supporting Information S1

## Data Availability

The information of 204 studies included in this review paper were listed in Supplementary Table 1 in Supporting Information S1 in the paper and in the reference list. Data and Stata code for reproducible tables are available at https://osf.io/vqsje/.
