# Supplementary material for: Effects of Desert Dust and Sandstorms on Human Health: A Scoping Review
Source: Geohealth. 2023 Mar 1;7(3):e2022GH000728. doi: 10.1029/2022GH000728 (PMC9976568; doi:10.1029/2022GH000728)
Supplement: Supplementary file 1 — Supporting Information S1 [file GH2-7-e2022GH000728-s001.docx]

**Effects of desert dust and sandstorms on human health: a scoping review**

**Supplementary Material**

Appendix A. Literature search strategy…………………………………………………………. 2

Supplementary Table 1. Characteristics, methods, and health outcomes of studies included in the scoping review ………………………………………………………………………………….. 3

Supplementary Table 2. List of countries in which studies for health effects of desert dust have been conducted ………………………………………………………………………………... 16

Supplementary Table 3. Health outcomes studied by desert dust exposure metric …………... 17

Supplementary Table 4. Health conditions studied by desert dust exposure metric ………….. 18

Supplementary Table 5. Health effects reported by desert dust exposure metric …………….. 19

Supplementary Table 6. Health outcomes by health conditions studied ……………………… 20

Supplementary Table 7. Health effects reported by health outcomes studied …………...…… 21

Supplementary Table 8. Health effects reported by health conditions studied. ………………. 22

Supplementary Figure 1. Number of studies included per year ………………………………. 23

Supplementary Figure 2. Main sources of desert dust in the world …………………...……… 24

List of studies included in the scoping review ………………………………………………... 25

**Appendix A. Literature search strategy.**

- **PubMed search strategy:**

((Desert dust[Title/Abstract] OR Sand storm*[Title/Abstract] OR Dust episode*[Title/Abstract] OR Dust event*[Title/Abstract] OR Dust outbreak*[Title/Abstract]) OR (Saharan dust[Title/Abstract] OR Saharan desert dust[Title/Abstract] OR African dust[Title/Abstract] OR African desert dust[Title/Abstract] OR Calima[Title/Abstract]) OR (Asian dust[Title/Abstract] OR Asian desert dust[Title/Abstract] OR Yellow dust[Title/Abstract] OR Yellow desert dust[Title/Abstract] OR Yellow sand[Title/Abstract] OR Yellow desert sand[Title/Abstract] OR Kosa[Title/Abstract] OR Gobi[Title/Abstract])

OR (Middle East dust[Title/Abstract] OR Middle East desert dust[Title/Abstract])) AND (health OR mortality OR hospital* OR emergency OR admission* OR clinic OR visit* OR ambulance* OR case series OR primary care OR symptom* OR disease*)

- **Web of Science search strategy:**

TS=(desert dust OR sand storm OR dust episode OR dust event OR dust outbreak OR saharan dust OR african dust OR asian dust OR yellow dust OR middle east dust OR colima OR kosa OR gobi) AND TS=(health OR mortality OR hospital* OR emergency OR admission* OR clinic OR visit* OR ambulance OR case series OR primary care OR symptom* OR disease*)

*refined document type by article and review article

- **Scopus search strategy:**

TITLE-ABS-KEY ( "desert dust" OR "sand storm" OR "dust episode" OR "dust event" OR "dust event" OR "dust outbreak" OR "saharan dust" OR "african dust" OR "asian dust" OR "yellow dust" OR "middle east dust" OR calima OR kosa OR gobi ) AND DOCTYPE ( ar OR re ) AND SRCTYPE ( j ) AND LANGUAGE ( english ) AND ALL ( health OR mortality OR hospital OR emergency OR admission OR clinic OR visit OR ambulance OR "case series" OR "primary care" OR symptom OR disease )

**Supplementary Table 1. Characteristics, methods, and health outcomes of studies included in the scoping review.**

| **(First author,**  **year of publication)** | **Country** | **Study**  **period** | **Study**  **design** | **Study**  **population** | **Health**  **outcome** | **Health**  **condition** | **Desert**  **origin** | **Desert dust**  **exposure method** | **Desert dust**  **exposure metric** | **Health**  **effect** |
| --- | --- | --- | --- | --- | --- | --- | --- | --- | --- | --- |
| (Kwon, Cho et al. 2002) | South Korea | 1995-1998 | Time-series | <65, ≥65 | Mortality | All causes, Cardiovascular, Respiratory | Asian | Not reported | Binary (risk factor) | Non-significant |
| (Chen, Sheen et al. 2004) | Taiwan | 1995-2000 | Time-series | all ages | Mortality | Cardiovascular, Respiratory | Asian | Threshold value | Binary (risk factor) | Non-significant |
| (Chen and Yang 2005) | Taiwan | 1996-2001 | Time-series | all ages | Hospital admissions or visits | Cardiovascular | Asian | Modelling | Binary (risk factor) | Non-significant |
| (Park, Lim et al. 2005) | South Korea | 2002 | Longitudinal | 16-75 | Symptoms and dysfunction | Respiratory | Asian | Visibility | Binary (risk factor) | Adverse |
| (Yang 2006) | Taiwan | 1996-2001 | Time-series | all ages | Hospital admissions or visits | Respiratory | Asian | Threshold value | Binary (risk factor) | Non-significant |
| (Yang, Chen et al. 2005) | Taiwan | 1996-2001 | Time-series | all ages | Hospital admissions or visits | Cerebrovascular | Asian | Threshold value | Binary (risk factor) | Adverse |
| (Yang, Tsai et al. 2005) | Taiwan | 1996-2001 | Time-series | all ages | Hospital admissions or visits | Cardiovascular | Asian | Threshold value | Binary (risk factor) | Non-significant |
| (Chang, Lee et al. 2006) | Taiwan | 1997-2001 | Time-series | all ages | Hospital admissions or visits | Allergic diseases | Asian | Threshold value | Binary (risk factor) | Non-significant |
| (Yang, Cheng et al. 2009) | Taiwan | 1997-2001 | Time-series | all ages | Hospital admissions or visits | Infectious diseases | Asian | Threshold value | Binary (risk factor) | Non-significant |
| (Bennett, McKendry et al. 2006) | Canada | 1997-1999 | Time-series | all ages | Hospital admissions or visits | Cardiovascular, Respiratory | Asian | Modelling | Binary (risk factor), Binary (effect modifier) | Non-significant |
| (Lee, Son et al. 2007) | South Korea | 2000-2004 | Time-series | all ages | Mortality | All causes | Asian | Multi-tool approach | Binary (effect modifier) | Adverse |
| (Meng and Lu 2007) | China | 1994–2003 | Time-series | all ages | Hospital admissions or visits | Cardiovascular, Respiratory | Asian | Visibility | Binary (risk factor) | Adverse |
| (Bell, Levy et al. 2008) | Taiwan | 1995-2002 | Time-series | all ages | Hospital admissions or visits | Cardiovascular, Cerebrovascular, Respiratory | Asian | Threshold value | Binary (risk factor) | Adverse |
| (Chan, Chuang et al. 2008) | Taiwan | 1995-2002 | Time-series | all ages | Emergency room visits | Cardiovascular, Respiratory | Asian | Modelling | Binary (risk factor) | Adverse |
| (Cheng, Ho et al. 2008) | Taiwan | 1996-2001 | Time-series | all ages | Hospital admissions or visits | Respiratory | Asian | Modelling | Binary (risk factor) | Adverse |
| (Chiu, Tiao et al. 2008) | Taiwan | 1996-2001 | Time-series | all ages | Hospital admissions or visits | Respiratory | Asian | Threshold value | Binary (risk factor) | Non-significant |
| (Lai and Cheng 2008) | Taiwan | 2000-2004 | Time-series | 0-4, 5-14, 15-64, ≥65 | Hospital admissions or visits | Respiratory | Asian | Threshold value | Binary (risk factor) | Adverse |
| (Yoo, Choung et al. 2008) | South Korea | 2004 | Time-series | children | Symptoms and dysfunction | Respiratory | Asian | Multi-tool approach | Binary (risk factor) | Adverse |
| (Kanatani, Ito et al. 2010) | Japan | 2005-2009 | Case-crossover | 1-5 | Hospital admissions or visits | Respiratory | Asian | Remote sensing | Binary (risk factor) | Adverse |
| (Mu, Battsetseg et al. 2010) | Mongolia | 2008 | Cross-sectional | 20-65 | Symptoms and dysfunction | Respiratory, Allergic skin and eye problems, Health related quality of life | Asian | Not reported | Binary (risk factor) | Non-significant |
| (Ueda, Nitta et al. 2010) | Japan | 2001-2007 | Case-crossover | <12 | Hospital admissions or visits | Respiratory | Asian | Visibility | Binary (risk factor) | Non-significant |
| (Hong, Pan et al. 2010) | South Korea | 2007 | Time-series | 9 | Symptoms and dysfunction | Respiratory | Asian | Multi-tool approach | Continuous risk factor | Non-significant |
| (Watanabe, Igishi et al. 2011) | Japan | 2009 | Longitudinal | ≥18 | Symptoms and dysfunction | Respiratory | Asian | Multi-tool approach | Binary (effect modifier) | Non-significant |
| (Watanabe, Yamasaki et al. 2011) | Japan | 2007 | Longitudinal | ≥18 | Symptoms and dysfunction | Respiratory | Asian | Multi-tool approach | Binary (effect modifier) | Adverse |
| (Chan and Ng 2011) | Taiwan | 1994-2007 | Case-crossover | all ages | Mortality | All causes, Cardiovascular, Respiratory | Asian | Multi-tool approach | Binary (risk factor) | Adverse |
| (Mu, Battsetseg et al. 2011) | Mongolia | 2008 | Cross-sectional | all ages | Symptoms and dysfunction | Respiratory, Allergic skin and eye problems | Asian | Not reported | Binary (risk factor) | Not applicable |
| (Otani, Onishi et al. 2011) | Japan | 2009 | Time-series | adults | Symptoms and dysfunction | Respiratory, Allergic skin and eye problems | Asian | Visibility | Binary (risk factor) | Adverse |
| (Watanabe, Kurai et al. 2012) | Japan | 2007-2011 | Longitudinal | ≥18 | Symptoms and dysfunction | Respiratory | Asian | Multi-tool approach | Binary (effect modifier) | Non-significant |
| (Chien, Yang et al. 2012) | Taiwan | 1997-2007 | Time-series | ≤14 | Hospital admissions or visits | Respiratory | Asian | Threshold value | Binary (risk factor) | Adverse |
| (Gupta, Singh et al. 2012) | India | not reported | Experimental | all ages | Symptoms and dysfunction | Respiratory | Asian | Threshold value | Binary (risk factor) | Adverse |
| (Kamouchi, Ueda et al. 2012) | Japan | 1999-2010 | Case-crossover | ≥20 | Hospital admissions or visits | Cardiovascular | Asian | Visibility | Binary (risk factor) | Adverse |
| (Kang, Keller et al. 2012) | Taiwan | 2000-2009 | Time-series | 0-6, 7-17, 18-44, 45-64, ≥65 | Hospital admissions or visits | Respiratory | Asian | Multi-tool approach | Binary (risk factor) | Adverse |
| (Kim, Kim et al. 2012) | South Korea | 2003-2006 | Time-series | all ages, ≥75 | Mortality | All causes, Cardiovascular | Asian | Threshold value | Binary (risk factor) | Adverse |
| (Onishi, Kurosaki et al. 2012) | Japan | 2009 | Cross-sectional | adults | Symptoms and dysfunction | Respiratory, Allergic skin and eye problems | Asian | Multi-tool approach | Binary (risk factor) | Adverse |
| (Otani, Onishi et al. 2012) | Japan | 2010 | Case-control | adults | Symptoms and dysfunction | Allergic skin and eye problems | Asian | Visibility | Binary (risk factor) | Adverse |
| (Tam, Wong et al. 2012) | Hong Kong | 1998-2002 | Case-crossover | all ages | Hospital admissions or visits | Respiratory, Infectious diseases | Asian | Multi-tool approach | Binary (risk factor) | Adverse |
| (Tam, Wong et al. 2012) | Hong Kong | 1998-2002 | Case-crossover | all ages | Hospital admissions or visits | Cardiovascular, Cerebrovascular | Asian | Multi-tool approach | Binary (risk factor) | Adverse |
| (Tao, An et al. 2012) | China | 2001-2005 | Time-series | <65, ≥65 | Hospital admissions or visits | Respiratory | Asian | Not reported | Binary (risk factor) | Adverse |
| (Ueda, Shimizu et al. 2012) | Japan | 2003-2007 | Case-crossover | ≥20 | Ambulance transports | All causes, Cardiovascular, Respiratory | Asian | Multi-tool approach | Binary (risk factor) | Adverse |
| (Yu, Yang et al. 2012) | Taiwan | 1997-2007 | Time-series | 0-6, 7-14 | Hospital admissions or visits | Respiratory | Asian | Multi-tool approach | Binary (risk factor) | Adverse |
| (Kashima, Yorifuji et al. 2012) | Japan | 2005-2010 | Time-series | ≥65 | Mortality | All causes | Asian | Remote sensing | Continuous (two sources) | Adverse |
| (Kang, Liu et al. 2013) | Taiwan | 2000-2009 | Time-series | ≥18 | Hospital admissions or visits | Cardiovascular | Asian | Threshold value | Binary (risk factor) | Adverse |
| (Lee, Kim et al. 2013) | South Korea | 2001-2009 | Time-series | <65, ≥65 | Mortality | All causes, Cardiovascular, Respiratory | Asian | Multi-tool approach | Binary (risk factor) | Adverse |
| (Yu, Chien et al. 2013) | Taiwan | 1998-2007 | Time-series | 0-6, 7-14 | Hospital admissions or visits | Respiratory | Asian | Threshold value | Binary (risk factor) | Adverse |
| (Higashi, Kambayashi et al. 2014) | Japan | 2011 | Time-series | adults | Symptoms and dysfunction | Respiratory | Asian | Remote sensing | Binary (effect modifier) | Adverse |
| (Watanabe, Kurai et al. 2014) | Japan | 2011 | Longitudinal | ≥18 | Symptoms and dysfunction | Respiratory | Asian | Multi-tool approach | Binary (effect modifier) | Adverse |
| (Watanabe, Kurai et al. 2014) | Japan | 2011 | Longitudinal | ≥18 | Symptoms and dysfunction | Respiratory | Asian | Multi-tool approach | Binary (effect modifier) | Adverse |
| (Chien, Lien et al. 2014) | Taiwan | 2002-2007 | Time-series | ≤14 | Hospital admissions or visits | Infectious diseases | Asian | Threshold value | Binary (risk factor) | Adverse |
| (Higashi, Kambayashi et al. 2014) | Japan | 2011 | Time-series | adults | Symptoms and dysfunction | Respiratory | Asian | Remote sensing | Binary (risk factor) | Adverse |
| (Lee and Lee 2014) | South Korea | 2005-2008 | Time-series | all ages | Hospital admissions or visits | Respiratory | Asian | Multi-tool approach | Binary (risk factor) | Adverse |
| (Lee, Honda et al. 2014) | South Korea, Taiwan, Japan | 2001-2009 | Time-series | <65, ≥65 | Mortality | All causes, Cardiovascular, Respiratory | Asian | Multi-tool approach | Binary (risk factor) | Adverse |
| (Matsukawa, Michikawa et al. 2014) | Japan | 2003-2010 | Case-crossover | ≥20 | Hospital admissions or visits | Cardiovascular | Asian | Visibility | Binary (risk factor) | Adverse |
| (Mimura, Yamagami et al. 2014) | Japan | 2011 | Cross-sectional | <20 | Symptoms and dysfunction | Allergic diseases | Asian | Source appointment | Binary (risk factor) | Adverse |
| (Otani, Onishi et al. 2014) | Japan | 2012 | Longitudinal | all ages | Symptoms and dysfunction | Respiratory, Allergic skin and eye problems | Asian | Visibility | Binary (risk factor) | Adverse |
| (Wang, Chen et al. 2014) | Taiwan | 2000-2009 | Time-series | 0-6, 7-17, 18-44, 45-74, ≥75 | Hospital admissions or visits | Respiratory | Asian | Threshold value | Binary (risk factor) | Adverse |
| (Kashima, Yorifuji et al. 2014) | Japan | 2006-2010 | Time-series | ≥65 | Ambulance transports | All causes, Cardiovascular, Cerebrovascular, Respiratory | Asian | Remote sensing | Continuous (two sources) | Adverse |
| (Watanabe, Kurai et al. 2015) | Japan | 2012 | Longitudinal | ≥18 | Symptoms and dysfunction | Respiratory | Asian | Multi-tool approach | Binary (effect modifier) | Adverse |
| (Aili and Oanh 2015) | China | 2013 | Time-series | all ages | Symptoms and dysfunction | Respiratory, Cardiovascular, Allergic skin and eye problems | Asian | Visibility | Binary (risk factor) | Adverse |
| (Nakamura, Hashizume et al. 2015) | Japan | 2005-2008 | Case-crossover | all ages | Ambulance transports | Cardiovascular | Asian | Multi-tool approach | Binary (risk factor) | Non-significant |
| (Park, Lim et al. 2015) | South Korea | 2006-2012 | Case-crossover | all ages | Hospital admissions or visits | Respiratory | Asian | Multi-tool approach | Binary (risk factor) | Adverse |
| (Wang and Lin 2015) | Taiwan | 2000-2008 | Time-series | all ages, ≥65 | Mortality | All causes, Cardiovascular, Respiratory | Asian | Multi-tool approach | Binary (risk factor) | Adverse |
| (Wang, Li et al. 2015) | China | 2011 | Case-control | ≥40 | Symptoms and dysfunction | Respiratory | Asian | Multi-tool approach | Binary (risk factor) | Adverse |
| (Watanabe, Noma et al. 2015) | Japan | 2012-2013 | Longitudinal | 44782 | Symptoms and dysfunction | Respiratory, Allergic skin and eye problems | Asian | Remote sensing | Binary (risk factor) | Adverse |
| (Onishi, Otani et al. 2015) | Japan | 2009 | Longitudinal | adults | Symptoms and dysfunction | Respiratory, Allergic skin and eye problems | Asian | Multi-tool approach | Not applicable | Adverse |
| (Watanabe, Noma et al. 2015) | Japan | 2013 | Longitudinal | ≥18 | Symptoms and dysfunction | Respiratory | Asian | Remote sensing | Not applicable | Adverse |
| (Ma, Xiao et al. 2016) | China | 2007-2011 | Time-series | 0-16, 16-40, 40-60, ≥60 | Emergency room visits | Respiratory | Asian | Visibility | Binary (effect modifier) | Adverse |
| (Majbauddin, Onishi et al. 2016) | Japan | 2013 | Longitudinal | all ages | Symptoms and dysfunction | Respiratory, Allergic skin and eye problems | Asian | Visibility | Binary (effect modifier) | Adverse |
| (Kanatani, Hamazaki et al. 2016) | Japan | 2011-2013 | Cohort | pregnant women and their children | Symptoms and dysfunction | Allergic diseases | Asian | Remote sensing | Binary (risk factor) | Adverse |
| (Ko, Hayashi et al. 2016) | Japan | 2013 | Cross-sectional | adults | Symptoms and dysfunction | Allergic skin and eye problems | Asian | Source appointment | Binary (risk factor) | Adverse |
| (Lin, Chen et al. 2016) | Taiwan | 2000-2008 | Time-series | all ages | Hospital admissions or visits | All causes, Cardiovascular, Respiratory | Asian | Multi-tool approach | Binary (risk factor) | Adverse |
| (Nakamura, Hashizume et al. 2016) | Japan | 2010-2013 | Case-crossover | 0-15 | Emergency room visits | Respiratory | Asian | Multi-tool approach | Binary (risk factor) | Adverse |
| (Park, Kim et al. 2016) | South Korea | 2007-2013 | Case-crossover | all ages | Hospital admissions or visits | Respiratory | Asian | Not reported | Binary (risk factor) | Adverse |
| (Teng, Chan et al. 2016) | Taiwan | 2000-2009 | Time-series | <45, 45-64, 65-74, ≥75 | Hospital admissions or visits | Cardiovascular | Asian | Threshold value | Binary (risk factor) | Adverse |
| (Wang, Wang et al. 2016) | China | 2005-2012 | Time-series | all ages | Hospital admissions or visits | Infectious diseases | Asian | Modelling | Binary (risk factor) | Adverse |
| (Kashima, Yorifuji et al. 2016) | South Korea, Japan | 2005-2011 | Time-series | ≥65 | Mortality | All causes | Asian | Remote sensing | Continuous (two sources) | Adverse |
| (Watanabe, Noma et al. 2016) | Japan | 2012 | Longitudinal | 44782 | Symptoms and dysfunction | Respiratory, Allergic skin and eye problems | Asian | Remote sensing | Not applicable | Adverse |
| (Watanabe, Noma et al. 2016) | Japan | 2012 | Longitudinal | ≥18 | Symptoms and dysfunction | Respiratory | Asian | Remote sensing | Not applicable | Adverse |
| (Watanabe, Noma et al. 2016) | Japan | 2012 | Longitudinal | ≥18 | Symptoms and dysfunction | Respiratory | Asian | Remote sensing | Not applicable | Adverse |
| (Liu, Liao et al. 2017) | Taiwan | 2006-2008 | Case-crossover | <65, ≥65 | Emergency room visits | Cardiovascular, Respiratory | Asian | Multi-tool approach | Binary (effect modifier) | Adverse |
| (Ma, Zhou et al. 2017) | China | 2007-2011 | Time-series | 0-44, 45-59, ≥60 | Hospital admissions or visits | Cardiovascular | Asian | Visibility | Binary (effect modifier) | Adverse |
| (Wong, Ho et al. 2017) | Hong Kong | 2009-2010 | Time-series | all ages | Mortality | All causes | Asian | Not reported | Binary (effect modifier) | Adverse |
| (Kojima, Michikawa et al. 2017) | Japan | 2010-2015 | Case-crossover | 20-64, 65-74, ≥75 | Symptoms and dysfunction | Cardiovascular | Asian | Visibility | Binary (risk factor) | Adverse |
| (Kurai, Watanabe et al. 2017) | Japan | 2013 | Longitudinal | adults ≥18, school children 9-10 | Symptoms and dysfunction | Respiratory | Asian | Remote sensing | Binary (risk factor) | Adverse |
| (Ma, Zhang et al. 2017) | China | 1965-2005 | Time-series | all ages | Mortality | Infectious diseases | Asian | Multi-tool approach | Binary (risk factor) | Adverse |
| (Sakata, Konishi et al. 2017) | Japan | 1989-2012 | Time-series | all ages | Hospital admissions or visits | Allergic diseases | Asian | Multi-tool approach | Binary (risk factor) | Adverse |
| (Kashima, Yorifuji et al. 2017) | Japan | 2006-2010 | Case-crossover | ≥65 | Ambulance transports | Cardiovascular, Cerebrovascular, Respiratory | Asian | Remote sensing | Continuous (two sources) | Adverse |
| (Altindag, Baek et al. 2017) | South Korea | 2003-2011 | Time-series | children | Mortality | Not specified | Asian | Not reported | Continuous risk factor | Adverse |
| (Watanabe, Noma et al. 2017) | Japan | 2015 | Longitudinal | 44846 | Symptoms and dysfunction | Allergic skin and eye problems | Asian | Remote sensing | Not applicable | Non-significant |
| (Nakao, Ishihara et al. 2018) | South Korea | 2013-2015 | Longitudinal | 40-79 | Symptoms and dysfunction | Respiratory, Health related quality of life | Asian | Multi-tool approach | Binary (confounder) | Adverse |
| (Ho, Wong et al. 2018) | Hong Kong | 2006-2010 | Case-crossover | all ages, ≥65 | Mortality | All causes, Cardiovascular, Respiratory | Asian | Multi-tool approach | Binary (effect modifier) | Adverse |
| (Chan, Teng et al. 2018) | Taiwan | 2000-2009 | Time-series | all ages | Hospital admissions or visits | Diabetes | Asian | Threshold value | Binary (risk factor) | Adverse |
| (Li, Chen et al. 2018) | China | 2010-2014 | Quasi-experimental | 44849 | Symptoms and dysfunction | Mental health | Asian | Visibility | Binary (risk factor) | Adverse |
| (Byun, Kim et al. 2019) | South Korea | 1998-2015 | Case-crossover | all ages | Mortality | All causes, Cardiovascular, Respiratory | Asian | Multi-tool approach | Binary (effect modifier) | Adverse |
| (Ng, Hashizume et al. 2019) | Japan | 2014-2016 | Longitudinal | 20-65 | Symptoms and dysfunction | Respiratory | Asian | Visibility | Binary (effect modifier) | Adverse |
| (Lee, Jung et al. 2019) | South Korea | 2002-2015 | Case-crossover | <35,35-64, ≥65 | Mortality | Mental health | Asian | Multi-tool approach | Binary (risk factor) | Adverse |
| (Nakao, Yamauchi et al. 2019) | Japan | 2010-2015 | Longitudinal | 40-79 | Symptoms and dysfunction | Respiratory, Health related quality of life | Asian | Not reported | Binary (risk factor) | Adverse |
| (Nakao, Yamauchi et al. 2019) | Japan | 2010-2015 | Longitudinal | 50-79 | Symptoms and dysfunction | Respiratory, Health related quality of life | Asian | Not reported | Binary (risk factor) | Adverse |
| (Lien, Owili et al. 2019) | Asia | 2000-2015 | Cross-sectional | pregnant women and their children | Symptoms and dysfunction | Under-5 mortality, Maternity and reproduction | Asian | Multi-tool approach | Not applicable | Adverse |
| (Ishii, Seki et al. 2020) | Japan | 2012-2016 | Case-crossover | all ages | Hospital admissions or visits | Respiratory | Asian | Visibility | Binary (risk factor) | Adverse |
| (Ishii, Seki et al. 2020) | Japan | 2005-2015 | Cross-sectional | 20-30, 31-40, 41-50, 51-60, ≥61 | Symptoms and dysfunction | Cardiovascular | Asian | Visibility | Binary (risk factor) | Adverse |
| (Michikawa, Yamazaki et al. 2020) | Japan | 2009-2014 | Case-crossover | adults | Hospital admissions or visits | Adverse birth outcomes | Asian | Multi-tool approach | Binary (risk factor) | Adverse |
| (Nakamura, Nishiwaki et al. 2020) | Japan | 2014-2016 | Case-crossover | children | Symptoms and dysfunction | Respiratory, Allergic skin and eye problems | Asian | Multi-tool approach | Binary (risk factor) | Adverse |
| (Itazawa, Kanatani et al. 2020) | Japan | 2014-2016 | Longitudinal | children | Symptoms and dysfunction | Respiratory, Allergic skin and eye problems | Asian | Remote sensing | Continuous (two sources) | Adverse |
| (Chen, Chan et al. 2021) | Taiwan | 2000-2012 | Time-series | all ages | Hospital admissions or visits | Respiratory | Asian | Threshold value | Binary (risk factor) | Adverse |
| (Hasunuma, Takeuchi et al. 2021) | Japan | 2013 | Case-crossover | 6-12 | Symptoms and dysfunction | Respiratory | Asian | Multi-tool approach | Binary (risk factor) | Adverse |
| (Lee, Lee et al. 2021) | South Korea | 2005-2017 | Time-series | <65, ≥65 | Emergency room visits | Cardiovascular | Asian | Visibility | Binary (risk factor) | Adverse |
| (Watanabe, Noma et al. 2021) | Japan | 2016-2018 | Case-crossover | children | Symptoms and dysfunction | School absence due to sickness | Asian | Remote sensing | Continuous (two sources) | Adverse |
| (Gyan, Henry et al. 2005) | Trinidad | 2001-2002 | Time-series | ≤15 | Emergency room visits | Respiratory | African | Visibility | Not applicable | Adverse |
| (Middleton, Yiallouros et al. 2008) | Cyprus | 1995-2004 | Time-series | 0-14, ≥15 | Hospital admissions or visits | Cardiovascular, Respiratory | African | Threshold value | Binary (effect modifier) | Adverse |
| (Perez, Tobias et al. 2008) | Spain | 2003-2004 | Case-crossover | <75, ≥75 | Mortality | All causes | African | Multi-tool approach | Binary (effect modifier) | Adverse |
| (Prospero, Blades et al. 2008) | Barbados | 1996-1997 | Time-series | ≤18 | Hospital admissions or visits | Respiratory | African | Source appointment | Binary (risk factor), Binary (effect modifier) | Non-significant |
| (Jiménez, Linares et al. 2010) | Spain | 2003-2005 | Time-series | ≥75 | Mortality | All causes, Cardiovascular, Respiratory | African | Remote sensing | Binary (effect modifier) | Adverse |
| (Dogan, Saydam et al. 2010) | Turkey | 2009 | Time-series | all ages | Hospital admissions or visits | Respiratory | African | Threshold value | Not applicable | Adverse |
| (Mallone, Stafoggia et al. 2011) | Italy | 2001-2004 | Case-crossover | ≥35 | Mortality | All causes, Cardiovascular, Cerebrovascular, Respiratory | African | Remote sensing | Binary (effect modifier) | Adverse |
| (Samoli, Kougea et al. 2011) | Greece | 2001-2006 | Time-series | <75, ≥75 | Mortality | All causes, Cardiovascular, Respiratory | African | Multi-tool approach | Binary (effect modifier) | Adverse |
| (Samoli, Nastos et al. 2011) | Greece | 2001-2004 | Time-series | 0-4, 5-14 | Hospital admissions or visits | Respiratory | African | Multi-tool approach | Binary (effect modifier) | Adverse |
| (Tobías, Pérez et al. 2011) | Spain | 2003-2005 | Case-crossover | all ages, <65, 65-74, ≥75 | Mortality | All causes | African | Multi-tool approach | Binary (effect modifier) | Adverse |
| (Dadvand, Basagaña et al. 2011) | Spain | 2003-2005 | Cohort | pregnant women and their children | Symptoms and dysfunction | Maternity and reproduction | African | Multi-tool approach | Binary (risk factor) | Non-significant |
| (Nastos, Kampanis et al. 2011) | Greece | 2008 | Time-series | all ages | Hospital admissions or visits | Cardiovascular, Respiratory | African | Multi-tool approach | Binary (risk factor) | Adverse |
| (Tobías, Caylà et al. 2011) | Spain | 2002-2009 | Time-series | all ages | Symptoms and dysfunction | Infectious diseases | African | Multi-tool approach | Binary (risk factor) | Adverse |
| (Zauli Sajani, Miglio et al. 2011) | Italy | 2002-2006 | Case-crossover | ≥75 | Mortality | All causes, Cardiovascular, Respiratory | African | Modelling | Binary (risk factor), Binary (effect modifier) | Adverse |
| (Díaz, Tobías et al. 2012) | Spain | 2003-2005 | Case-crossover | all ages | Mortality | Cardiovascular, Cerebrovascular, Respiratory | African | Multi-tool approach | Binary (effect modifier) | Adverse |
| (Perez, Tobías et al. 2012) | Spain | 2003-2007 | Case-crossover | all ages | Mortality | Cardiovascular, Cerebrovascular, Respiratory | African | Multi-tool approach | Binary (effect modifier) | Adverse |
| (Perez, Tobías et al. 2012) | Spain | 2003-2007 | Time-series | all-ages | Mortality | Cardiovascular | African | Multi-tool approach | Continuous (three sources) | Adverse |
| (Alessandrini, Stafoggia et al. 2013) | Italy | 2001-2004 | Time-series | <14, ≥35 | Hospital admissions or visits | Cardiovascular, Cerebrovascular, Respiratory | African | Remote sensing | Binary (effect modifier) | Adverse |
| (Meo, Al-Kheraiji et al. 2013) | Saudi Arabia | 2011-2012 | Cross-sectional | all ages | Symptoms and dysfunction | Respiratory, Allergic skin and eye problems | African | Not reported | Binary (risk factor) | Adverse |
| (Neophytou, Yiallouros et al. 2013) | Cyprus | 2004-2007 | Time-series | all ages | Mortality | All causes, Cardiovascular, Respiratory | African | Multi-tool approach | Binary (effect modifier) | Adverse |
| (Agier, Deroubaix et al. 2013) | Niger | 1986-2007 | Time-series | all ages | Symptoms and dysfunction | Infectious diseases | African | Multi-tool approach | Continuous risk factor | Non-significant |
| (Martiny and Chiapello 2013) | Niger, Mali | 2004-2009 | Time-series | all ages | Symptoms and dysfunction | Infectious diseases | African | Remote sensing | Not applicable | Adverse |
| (Cadelis, Tourres et al. 2014) | Guadeloupe | 2011 | Case-crossover | 5-15 | Emergency room visits | Respiratory | African | Multi-tool approach | Binary (effect modifier) | Adverse |
| (Pandolfi, Tobias et al. 2014) | Spain | 2003-2010 | Case-crossover | ≥65 | Mortality | All causes | African | Multi-tool approach | Binary (effect modifier) | Adverse |
| (Reyes, Díaz et al. 2014) | Spain | 2000-2003 | Time-series | all ages | Hospital admissions or visits | All causes, Cardiovascular, Respiratory | African | Multi-tool approach | Binary (effect modifier) | Adverse |
| (Vodonos, Friger et al. 2014) | Israel | 2001-2010 | Time-series | all ages, <50, 50-69, ≥70 | Hospital admissions or visits | Cardiovascular | African | Threshold value | Binary (risk factor) | Adverse |
| (Garcia-Pando, Stanton et al. 2014) | Niger | 1986-2006 | Time-series | all ages | Symptoms and dysfunction | Infectious diseases | African | Modelling | Continuous risk factor | Adverse |
| (De Longueville, Hountondji et al. 2014) | Benin | 2003-2007 | Time-series | <5 | Hospital admissions or visits | Respiratory | African | Multi-tool approach | Not applicable | Adverse |
| (Vodonos, Friger et al. 2015) | Israel | 2001-2010 | Case-crossover | all ages, ≥65 | Hospital admissions or visits | Cardiovascular | African | Threshold value | Binary (effect modifier) | Adverse |
| (Akpinar-Elci, Martin et al. 2015) | Grenada | 2001-2005 | Time-series | all ages | Hospital admissions or visits | Respiratory | African | Visibility | Continuous risk factor | Adverse |
| (Hayilu, Legesse et al. 2016) | Ethiopia | 2015 | Cross-sectional | ≥18 | Symptoms and dysfunction | Allergic skin and eye problems | African | Not reported | Binary (risk factor) | Adverse |
| (Stafoggia, Zauli-Sajani et al. 2016) | Europe | 2001-2010 | Case-crossover | 0-14, ≥15 | Mortality, Hospital admissions or visits | All causes, Cardiovascular, Respiratory | African | Multi-tool approach | Continuous (two sources) | Adverse |
| (Diokhane, Jenkins et al. 2016) | Senegal | 2012-2013 | Time-series | all ages | Symptoms and dysfunction | Infectious diseases | African | Remote sensing | Not applicable | Adverse |
| (Díaz, Linares et al. 2017) | Spain | 2004-2009 | Time-series | all ages | Mortality | All causes | African | Multi-tool approach | Binary (effect modifier) | Adverse |
| (Trianti, Samoli et al. 2017) | Greece | 2001-2006 | Time-series | 18-64, ≥65 | Hospital admissions or visits, Emergency room visits | Respiratory | African | Multi-tool approach | Binary (risk factor) | Adverse |
| (Menéndez, Derbyshire et al. 2017) | Spain | 2010-2011 | Time-series | 14-79, ≥80 | Hospital admissions or visits | Respiratory | African | Multi-tool approach | Not applicable | Non-significant |
| (Renzi, Forastiere et al. 2018) | Italy | 2006-2012 | Case-crossover | ≥35 | Mortality | All causes, Cardiovascular, Respiratory | African | Multi-tool approach | Continuous (two sources) | Adverse |
| (Woringer, Martiny et al. 2018) | Burkina Faso | 2004-2014 | Time-series | all ages | Symptoms and dysfunction | Infectious diseases | African | Remote sensing | Not applicable | Non-significant |
| (Lorentzou, Kouvarakis et al. 2019) | Greece | 2018 | Time-series | all ages | Hospital admissions or visits, Emergency room visits | Respiratory, Cardiovascular | African | Threshold value | Binary (risk factor) | Adverse |
| (Viel, Mallet et al. 2019) | Guadeloupe | 2005-2007 | Cohort | 20-34 | Hospital admissions or visits | Adverse birth outcomes | African | Multi-tool approach | Continuous risk factor | Adverse |
| (Augusto, Ratola et al. 2020) | Portugal | 2017-2018 | Time-series | all ages | Mortality | All causes, Cardiovascular, Respiratory | African | Modelling | Binary (effect modifier) | Adverse |
| (Dominguez-Rodriguez, Baez-Ferrer et al. 2020) | Spain | 2014-2017 | Time-series | all ages | Mortality | Cardiovascular | African | Threshold value | Binary (risk factor) | Adverse |
| (Dominguez-Rodriguez, Rodríguez et al. 2020) | Spain | 2017 | Cross-sectional | ≥50 | Symptoms and dysfunction | Cardiovascular | African | Multi-tool approach | Binary (risk factor) | Adverse |
| (Gutierrez, Zuidema et al. 2020) | United States of America | 2013-2016 | Cohort | all ages | Hospital admissions or visits | Respiratory | African | Remote sensing | Binary (risk factor) | Adverse |
| (Moreira, Linares et al. 2020) | Spain | 2004-2009 | Time-series | all ages | Symptoms and dysfunction | Adverse birth outcomes | African | Multi-tool approach | Binary (risk factor) | Adverse |
| (Tobías and Stafoggia 2020) | Italy | 2005-2015 | Time-series | all ages | Mortality | All causes | African | Multi-tool approach | Binary (risk factor), Binary (confounder), Binary (effect modifier), Continuous (two sources), Continuous (three sources) | Non-significant |
| (Heft-Neal, Burney et al. 2020) | Africa | 2001-2015 | Cross-sectional | all ages | Mortality | Under-5 mortality | African | Multi-tool approach | Not applicable | Adverse |
| (Karimi, Pouran et al. 2020) | Burkina Faso | 1993, 2003, 2010 | Cross-sectional | <5 | Mortality | Under-5 mortality | African | Threshold value | Not applicable | Adverse |
| (Viel, Michineau et al. 2020) | Guadeloupe | 2004-2007 | Cohort | 20-34 | Hospital admissions or visits | Adverse birth outcomes | African | Threshold value | Not applicable | Adverse |
| (Desalu, Adeoti et al. 2021) | Nigeria | 2017-2018 | Cross-sectional | ≥18 | Symptoms and dysfunction | Respiratory | African | Not reported | Binary (risk factor) | Adverse |
| (Domínguez-Rodríguez, Báez-Ferrer et al. 2021) | Spain | 2012-2017 | Case-crossover | all ages | Symptoms and dysfunction | Cardiovascular | African | Modelling | Binary (risk factor) | Non-significant |
| (Linares, Culqui et al. 2021) | Spain | 2020 | Time-series | all ages | Hospital admissions or visits | Infectious diseases | African | Multi-tool approach | Binary (risk factor) | Adverse |
| (López-Villarrubia, Costa Estirado et al. 2020) | Spain | 2001-2005 | Time-series | all ages | Emergency room visits | Respiratory | African | Multi-tool approach | Binary (risk factor) | Adverse |
| (Meo, Almutairi et al. 2021) | Saudi Arabia | 2021 | Cross-sectional | all ages | Mortality, Symptoms and dysfunction | Infectious diseases | African | Not reported | Binary (risk factor) | Adverse |
| (Saers, Andersson et al. 2021) | Mali | 2018-2019 | Cohort | adults | Symptoms and dysfunction | Respiratory | African | Source appointment | Binary (risk factor) | Adverse |
| (Silva, Fragoso et al. 2021) | Portugal | 2006-2015 | Time-series | 0-16, ≥65 | Hospital admissions or visits | Respiratory | African | Multi-tool approach | Binary (risk factor) | Adverse |
| (Bachwenkizi, Liu et al. 2021) | Africa | 2005-2015 | Cross-sectional | 0-11 months | Mortality | Under-5 mortality | African | Multi-tool approach | Not applicable | Adverse |
| (Al-Rifaia, Elmi et al. 2011) | Kuwait | 1998-2005 | Cross-sectional | all ages | Mortality | Not specified | Arabian | Not reported | Continuous risk factor | Non-significant |
| (Thalib and Al-Taiar 2012) | Kuwait | 1996-2000 | Time-series | 0-14, 15-64, ≥65 | Hospital admissions or visits | Respiratory | Arabian | Threshold value | Binary (risk factor) | Adverse |
| (Al-Taiar and Thalib 2014) | Kuwait | 1996-2000 | Time-series | all ages | Mortality | All causes, Cardiovascular, Respiratory | Arabian | Threshold value | Binary (risk factor) | Non-significant |
| (Ebrahimi, Ebrahimzadeh et al. 2014) | Iran | 2009-2010 | Time-series | all ages | Hospital admissions or visits | Cardiovascular, Respiratory | Arabian | Not reported | Binary (risk factor) | Adverse |
| (Gheybi, Movahed et al. 2014) | Iran | not reported | Case-control | all ages | Symptoms and dysfunction | Allergic diseases | Arabian | Threshold value | Binary (risk factor) | Adverse |
| (Ebenstein, Frank et al. 2015) | Israel | 2007-2009 | Time-series | all ages | Hospital admissions or visits | Respiratory | Arabian | Not reported | Binary (confounder) | Adverse |
| (Alangari, Riaz et al. 2015) | Saudi Arabia | 2012 | Time-series | 2-12 | Emergency room visits | Respiratory | Arabian | Threshold value | Binary (risk factor) | Non-significant |
| (Yitshak-Sade, Novack et al. 2015) | Israel | 2005-2011 | Time-series | <18 | Hospital admissions or visits | Respiratory | Arabian | Threshold value | Binary (risk factor) | Adverse |
| (Almasi, Bakhshi et al. 2016) | Iran | 2008-2013 | Time-series | all ages | Mortality, Symptoms and dysfunction | Accidents and injuries | Arabian | Not reported | Not applicable | Protective |
| (Geravandi, Sicard et al. 2017) | Iran | 2010-2012 | Time-series | all ages | Hospital admissions or visits | Respiratory | Arabian | Threshold value | Binary (risk factor) | Adverse |
| (Al-Hemoud, Al-Dousari et al. 2018) | Kuwait | 2012 | Time-series | all ages | Mortality, Symptoms and dysfunction | Cardiovascular, Respiratory | Arabian | Visibility | Binary (confounder) | Adverse |
| (Al, Bogan et al. 2018) | Turkey | 2009-2014 | Time-series | all ages | Mortality, Hospital admissions or visits, Emergency room visits | Cardiovascular | Arabian | Remote sensing | Binary (risk factor) | Adverse |
| (Aminharati, Dallal et al. 2018) | Iran | 2012-2015 | Time-series | all ages | Symptoms and dysfunction | Infectious diseases | Arabian | Threshold value | Binary (risk factor) | Adverse |
| (Al-Dousari, Ibrahim et al. 2018) | Kuwait | 2009-2011 | Time-series | all ages | Hospital admissions or visits | Respiratory | Arabian | Source appointment | Not applicable | Adverse |
| (Dastoorpoor, Idani et al. 2018) | Iran | 2008-2015 | Time-series | 18-35 | Hospital admissions or visits | Adverse birth outcomes, Maternity and reproduction | Arabian | Modelling | Not applicable | Adverse |
| (Radmanesh, Maleki et al. 2019) | Iran | 2012-2016 | Time-series | all ages | Hospital admissions or visits, Emergency room visits | Cerebrovascular | Arabian | Multi-tool approach | Binary (effect modifier) | Adverse |
| (Soleimani, Boloorani et al. 2019) | Iran | 2009-2015 | Time-series | all ages | Hospital admissions or visits | Cardiovascular | Arabian | Multi-tool approach | Binary (effect modifier) | Adverse |
| (Achilleos, Al-Ozairi et al. 2019) | Kuwait | 2000-2016 | Time-series | all ages | Mortality | All causes | Arabian | Visibility | Binary (risk factor) | Adverse |
| (Aminharati, Ehrampoush et al. 2019) | Iran | 2012-2016 | Cross-sectional | all ages | Symptoms and dysfunction | Infectious diseases | Arabian | Not reported | Binary (risk factor) | Adverse |
| (Aghababaeian, Dastoorpoor et al. 2019) | Iran | 2013-2016 | Time-series | all ages | Ambulance transports | Cardiovascular, Respiratory, Accidents and injuries | Arabian | Not reported | Not applicable | Adverse |
| (Shahsavani, Tobías et al. 2020) | Iran | 2014-2017 | Case-crossover | all ages | Mortality | All causes | Arabian | Threshold value | Binary (effect modifier) | Adverse |
| (Dallal, Ehrampoush et al. 2020) | Iran | 2012-2015 | Cross-sectional | all ages | Symptoms and dysfunction | Infectious diseases | Arabian | Threshold value | Binary (risk factor) | Adverse |
| (Aghababaeian, Ostadtaghizadeh et al. 2021) | Iran | 2014-2019 | Time-series | all ages | Mortality | All causes, Cardiovascular, Respiratory | Arabian | Threshold value | Binary (risk factor) | Adverse |
| (Bogan, Al et al. 2021) | Turkey | 2009-2014 | Time-series | adults | Symptoms and dysfunction | Adverse birth outcomes | Arabian | Remote sensing | Binary (risk factor) | Adverse |
| (Sadeghimoghaddam, Khankeh et al. 2021) | Iran | 2013-2020 | Cohort | all ages, ≥60 | Hospital admissions or visits | Cardiovascular | Arabian | Visibility | Binary (risk factor) | Adverse |
| (Baltaci, Arslan et al. 2022) | Turkey | 2007-2017 | Time-series | all ages | Hospital admissions or visits | Respiratory | Arabian | Multi-tool approach | Binary (risk factor) | Adverse |
| (Williams, Sable et al. 1979) | United States of America | 1977 | Case report | 15-62 | Hospital admissions or visits | Respiratory | American | Not reported | Binary (risk factor) | Adverse |
| (Schwartz, Norris et al. 1999) | United States of America | 1989-1995 | Case-control | all ages | Mortality | All causes | American | Threshold value | Binary (risk factor) | Non-significant |
| (Grineski, Staniswalis et al. 2011) | United States of America | 2011 | Case-crossover | 1-17, 18-64, ≥65 | Hospital admissions or visits | Respiratory | American | Modelling | Binary (risk factor) | Adverse |
| (Crooks, Cascio et al. 2016) | United States of America | 1993-2005 | Case-crossover | all ages | Mortality | All causes, Cardiovascular, Respiratory | American | Modelling | Binary (risk factor) | Adverse |
| (Tong, Wang et al. 2017) | United States of America | 2000-2011 | Cross-sectional | all ages | Symptoms and dysfunction | Infectious diseases | American | Remote sensing | Binary (risk factor) | Adverse |
| (Bhattachan, Okin et al. 2019) | United States of America | 2006-2016 | Time-series | all ages | Symptoms and dysfunction | Accidents and injuries | American | Multi-tool approach | Not applicable | Adverse |
| (Jones 2020) | United States of America | 2010-2017 | Cross-sectional | children | Symptoms and dysfunction | Adverse birth outcomes | American | Modelling | Binary (risk factor) | Adverse |
| (Herrera-Molina, Gill et al. 2021) | United States of America | 2010-2014 | Time-series | all ages | Hospital admissions or visits | All causes | American | Threshold value | Binary (confounder) | Adverse |
| (Comrie 2021) | United States of America | 2006-2020 | Time-series | all ages | Symptoms and dysfunction | Infectious diseases | American | Not reported | Binary (risk factor) | Non-significant |
| (Rublee, Sorensen et al. 2020) | United States of America | 2000-2015 | Case-crossover | all ages | Hospital admissions or visits | Cardiovascular, Respiratory | American, African | Multi-tool approach | Binary (risk factor) | Adverse |
| (Rutherford, Clark et al. 1999) | Australia | 1992-1994 | Time-series | all ages | Symptoms and dysfunction | Respiratory | Australian | Source appointment | Binary (risk factor) | Adverse |
| (Johnston, Hanigan et al. 2011) | Australia | 1997-2004 | Case-crossover | all ages | Mortality | All causes, Cardiovascular, Respiratory | Australian | Threshold value | Binary (risk factor) | Adverse |
| (Barnett, Fraser et al. 2012) | Australia | 2009 | Time-series | all ages | Hospital admissions or visits | All causes | Australian | Threshold value | Binary (risk factor) | Non-significant |
| (Merrifield, Schindeler et al. 2013) | Australia | 2004-2009 | Time-series | all ages, ≤5, ≥65 | Hospital admissions or visits, Emergency room visits | All causes, Cardiovascular, Respiratory | Australian | Not reported | Binary (risk factor) | Adverse |

**Supplementary Table 2. List of countries in which studies for health effects of desert dust have been conducted.**

| **Country by  desert dust source^*^** | **Num. studies** |
| --- | --- |
| **Asian desert** |  |
| Japan | 48 |
| Taiwan | 27 |
| South Korea | 17 |
| China | 9 |
| Hong Kong | 4 |
| Mongolia | 2 |
| Canada | 1 |
| India | 1 |
| Asia | 1 |
| **African desert** |  |
| Spain | 18 |
| Greece | 5 |
| Italy | 5 |
| Guadeloupe | 3 |
| Niger | 3 |
| Africa | 2 |
| Burkina Faso | 2 |
| Cyprus | 2 |
| Israel | 2 |
| Mali | 2 |
| Portugal | 2 |
| Saudi Arabia | 2 |
| United States of America | 2 |
| Barbados | 1 |
| Benin | 1 |
| Ethiopia | 1 |
| Grenada | 1 |
| Nigeria | 1 |
| Senegal | 1 |
| Trinidad | 1 |
| Turkey | 1 |
| Europe | 1 |
| **Arabian desert** |  |
| Iran | 14 |
| Kuwait | 6 |
| Turkey | 3 |
| Israel | 2 |
| Saudi Arabia | 1 |
| **American desert** |  |
| United States of America | 9 |
| **Australian desert** |  |
| Australia | 4 |

Note: ^*^One study examined both desert dust exposure from African and American sources.

**Supplementary Table 3. Health outcomes studied by desert dust exposure metric.**

|  | Dust exposure metric^*^ | | | | | | | | | | | | | |
| --- | --- | --- | --- | --- | --- | --- | --- | --- | --- | --- | --- | --- | --- | --- |
|  | Binary (risk factor) | | Binary (confounder) | | Binary (effect modifier) | | Continuous (two sources) | | Continuous (three sources) | | Continuous risk factor | | Not applicable | |
| Health outcome^*^ | n | (%) | n | (%) | n | (%) | n | (%) | n | (%) | n | (%) | n | (%) |
| Hospital admissions or visits | 56 | (42.8) | 2 | (33.3) | 10 | (24.4) | 1 | (10.0) | 0 | (0.0) | 2 | (28.6) | 6 | (25.0) |
| Symptoms and dysfunction | 43 | (32.8) | 2 | (33.3) | 9 | (22.0) | 2 | (20.0) | 0 | (0.0) | 3 | (42.9) | 12 | (50.0) |
| Mortality | 21 | (16.0) | 2 | (33.3) | 18 | (43.9) | 5 | (50.0) | 2 | (100.0) | 2 | (28.6) | 4 | (16.7) |
| Emergency room visits | 9 | (6.9) | 0 | (0.0) | 4 | (9.8) | 0 | (10.0) | 0 | (0.0) | 0 | (0.0) | 1 | (4.2) |
| Ambulance transports | 2 | (1.5) | 0 | (0.0) | 0 | (0.0) | 2 | (20.0) | 0 | (0.0) | 0 | (0.0) | 1 | (4.2) |

Note: ^*^Categories are not mutually exclusive.

**Supplementary Table 4.** **Health conditions studied by desert dust exposure metric.**

|  | Dust exposure metric^*^ | | | | | | | | | | | | | |
| --- | --- | --- | --- | --- | --- | --- | --- | --- | --- | --- | --- | --- | --- | --- |
|  | Binary (risk factor) | | Binary (confounder) | | Binary (effect modifier) | | Continuous (two sources) | | Continuous (three sources) | | Continuous risk factor | | Not applicable | |
| Health condition^*^ | n | (%) | n | (%) | n | (%) | n | (%) | n | (%) | n | (%) | n | (%) |
| Respiratory | 74 | (41.3) | 3 | (42.9) | 28 | (40.6) | 5 | (26.3) | 0 | (0.0) | 2 | (28.6) | 11 | (37.9) |
| Cardiovascular | 42 | (23.5) | 1 | (14.3) | 18 | (26.1) | 4 | (21.1) | 1 | (50.0) | 0 | (0.0) | 1 | (3.4) |
| All causes | 19 | (10.6) | 2 | (28.5) | 17 | (24.6) | 6 | (31.6) | 1 | (50.0) | 0 | (0.0) | 0 | (0.0) |
| Infectious diseases | 13 | (7.4) | 0 | (0.0) | 0 | (0.0) | 0 | (0.0) | 0 | (0.0) | 2 | (28.6) | 3 | (10.3) |
| Allergic skin and eye problems | 12 | (6.7) | 0 | (0.0) | 1 | (1.4) | 1 | (5.3) | 0 | (0.0) | 0 | (0.0) | 3 | (10.3) |
| Cerebrovascular | 3 | (1.7) | 0 | (0.0) | 5 | (7.2) | 2 | (10.5) | 0 | (0.0) | 0 | (0.0) | 0 | (0.0) |
| Adverse birth outcomes | 4 | (2.2) | 0 | (0.0) | 0 | (0.0) | 0 | (0.0) | 0 | (0.0) | 1 | (14.3) | 2 | (6.9) |
| Allergic diseases | 5 | (2.8) | 0 | (0.0) | 0 | (0.0) | 0 | (0.0) | 0 | (0.0) | 0 | (0.0) | 0 | (0.0) |
| Health related quality of life | 3 | (1.7) | 1 | (14.3) | 0 | (0.0) | 0 | (0.0) | 0 | (0.0) | 0 | (0.0) | 0 | (0.0) |
| Under-5 mortality | 0 | (0.0) | 0 | (0.0) | 0 | (0.0) | 0 | (0.0) | 0 | (0.0) | 0 | (0.0) | 4 | (13.8) |
| Accidents and injuries | 0 | (0.0) | 0 | (0.0) | 0 | (0.0) | 0 | (0.0) | 0 | (0.0) | 0 | (0.0) | 3 | (10.3) |
| Maternity and reproduction | 1 | (0.5) | 0 | (0.0) | 0 | (0.0) | 0 | (0.0) | 0 | (0.0) | 0 | (0.0) | 2 | (6.9) |
| Mental health | 2 | (1.1) | 0 | (0.0) | 0 | (0.0) | 0 | (0.0) | 0 | (0.0) | 0 | (0.0) | 0 | (0.0) |
| Diabetes | 1 | (0.5) | 0 | (0.0) | 0 | (0.0) | 0 | (0.0) | 0 | (0.0) | 0 | (0.0) | 0 | (0.0) |
| School absence due to sickness | 0 | (0.0) | 0 | (0.0) | 0 | (0.0) | 1 | (5.3) | 0 | (0.0) | 0 | (0.0) | 0 | (0.0) |
| Not specified | 0 | (0.0) | 0 | (0.0) | 0 | (0.0) | 0 | (0.0) | 0 | (0.0) | 2 | (28.6) | 0 | (0.0) |

Note: ^*^Categories are not mutually exclusive.

**Supplementary Table 5.** **Health effects reported by desert dust exposure metric.**

|  | Dust exposure metric^*^ | | | | | | | | | | | | | |
| --- | --- | --- | --- | --- | --- | --- | --- | --- | --- | --- | --- | --- | --- | --- |
|  | Binary (risk factor) | | Binary (confounder) | | Binary (effect modifier) | | Continuous (two sources) | | Continuous (three sources) | | Continuous risk factor | | Not applicable | |
| Health effect | n | (%) | n | (%) | n | (%) | n | (%) | n | (%) | n | (%) | n | (%) |
| Adverse | 104 | (83.2) | 35 | (87.5) | 8 | (88.9) | 8 | (88.9) | 1 | (50.0) | 4 | (57.1) | 19 | (82.6) |
| Non-significant | 20 | (16.0) | 5 | (12.5) | 1 | (11.1) | 1 | (11.1) | 1 | (50.0) | 3 | (42.9) | 3 | (13.0) |
| Protective | 0 | (0.0) | 0 | (0.0) | 0 | (0.0) | 0 | (0.0) | 0 | (0.0) | 0 | (0.0) | 1 | (4.3) |
| Not applicable | 1 | (0.8) | 0 | (0.0) | 0 | (0.0) | 0 | (0.0) | 0 | (0.0) | 0 | (0.0) | 0 | (0.0) |

Note: ^*^Categories are not mutually exclusive.

**Supplementary Table 6.** **Health outcomes by health conditions studied.**

|  | Health outcome^*^ | | | | | | | | | |
| --- | --- | --- | --- | --- | --- | --- | --- | --- | --- | --- |
|  | Hospital admissions or visits | | Mortality | | Symptoms and  dysfunction | | Emergency  room visits | | Ambulance  transports | |
| Health condition^*^ | n | (%) | n | (%) | n | (%) | n | (%) | n | (%) |
| Respiratory | 48 | (49.0) | 23 | (24.5) | 39 | (42.9) | 11 | (57.9) | 4 | (28.6) |
| Cardiovascular | 26 | (26.5) | 27 | (28.7) | 6 | (6.6) | 6 | (31.6) | 5 | (35.7) |
| All causes | 6 | (6.1) | 32 | (34.0) | 0 | (0.0) | 1 | (5.3) | 2 | (14.3) |
| Infectious diseases | 5 | (5.1) | 2 | (2.1) | 12 | (13.2) | 0 | (0.0) | 0 | (0.0) |
| Allergic skin and eye problems | 0 | (0.0) | 0 | (0.0) | 17 | (18.7) | 0 | (0.0) | 0 | (0.0) |
| Cerebrovascular | 5 | (5.1) | 3 | (3.2) | 0 | (0.0) | 1 | (5.3) | 2 | (14.3) |
| Adverse birth outcomes | 4 | (4.1) | 0 | (0.0) | 3 | (3.3) | 0 | (0.0) | 0 | (0.0) |
| Allergic diseases | 2 | (2.0) | 0 | (0.0) | 3 | (3.3) | 0 | (0.0) | 0 | (0.0) |
| Accidents and injuries | 0 | (0.0) | 1 | (1.1) | 2 | (2.2) | 0 | (0.0) | 1 | (7.1) |
| Health related quality of life | 0 | (0.0) | 0 | (0.0) | 4 | (4.4) | 0 | (0.0) | 0 | (0.0) |
| Under-5 mortality | 0 | (0.0) | 3 | (3.2) | 1 | (1.1) | 0 | (0.0) | 0 | (0.0) |
| Maternity and reproduction | 1 | (1.0) | 0 | (0.0) | 2 | (2.2) | 0 | (0.0) | 0 | (0.0) |
| Mental health | 0 | (0.0) | 1 | (1.1) | 1 | (1.1) | 0 | (0.0) | 0 | (0.0) |
| Diabetes | 1 | (1.0) | 0 | (0.0) | 0 | (0.0) | 0 | (0.0) | 0 | (0.0) |
| School absence due sickness | 0 | (0.0) | 0 | (0.0) | 1 | (1.1) | 0 | (0.0) | 0 | (0.0) |
| Not specified | 0 | (0.0) | 2 | (2.1) | 0 | (0.0) | 0 | (0.0) | 0 | (0.0) |

Note: ^*^Categories are not mutually exclusive.

**Supplementary** **Table 7. Health effects reported by health outcomes studied.**

|  | Health effect | | | | | | | |
| --- | --- | --- | --- | --- | --- | --- | --- | --- |
|  | Adverse | | Non-significant | | Protective | | Not applicable | |
| Health outcome^*^ | n | (%) | n | (%) | n | (%) | n | (%) |
| Hospital admissions or visits | 65 | (85.5) | 11 | (14.5) | 0 | (0.0) | 0 | (0.0) |
| Symptoms and dysfunction | 59 | (83.1) | 10 | (14.1) | 1 | (1.4) | 1 | (1.4) |
| Mortality | 41 | (85.4) | 6 | (12.5) | 1 | (2.1) | 0 | (0.0) |
| Emergency room visits | 13 | (92.9) | 1 | (7.1) | 0 | (0.0) | 0 | (0.0) |
| Ambulance transports | 4 | (80.0) | 1 | (20.0) | 0 | (0.0) | 0 | (0.0) |

Note: ^*^Categories are not mutually exclusive.

**Supplementary Table 8. Health effects reported by health conditions studied.**

|  | Health effect | | | | | | | |
| --- | --- | --- | --- | --- | --- | --- | --- | --- |
|  | Adverse | | Non-significant | | Protective | | Not applicable | |
| Health condition^*^ | n | (%) | n | (%) | n | (%) | n | (%) |
| Respiratory | 105 | (87.5) | 14 | (11.7) | 1 | (0.8) | 0 | (0.0) |
| Cardiovascular | 56 | (87.5) | 8 | (12.5) | 0 | (0.0) | 0 | (0.0) |
| All causes | 34 | (87.2) | 5 | (12.8) | 0 | (0.0) | 0 | (0.0) |
| Infectious diseases | 14 | (77.8) | 4 | (22.2) | 0 | (0.0) | 0 | (0.0) |
| Allergic skin and eye problems | 14 | (82.4) | 2 | (11.8) | 1 | (5.9) | 0 | (0.0) |
| Cerebrovascular | 10 | (100.0) | 0 | (0.0) | 0 | (0.0) | 0 | (0.0) |
| Adverse birth outcomes | 7 | (100.0) | 0 | (0.0) | 0 | (0.0) | 0 | (0.0) |
| Allergic diseases | 4 | (80.0) | 1 | (20.0) | 0 | (0.0) | 0 | (0.0) |
| Health related quality of life | 3 | (75.0) | 1 | (25.0) | 0 | (0.0) | 0 | (0.0) |
| Under-5 mortality | 4 | (100.0) | 0 | (0.0) | 0 | (0.0) | 0 | (0.0) |
| Accidents and injuries | 2 | (66.7) | 0 | (0.0) | 0 | (0.0) | 1 | (33.3) |
| Maternity and reproduction | 2 | (66.7) | 1 | (33.3) | 0 | (0.0) | 0 | (0.0) |
| Mental health | 2 | (100.0) | 0 | (0.0) | 0 | (0.0) | 0 | (0.0) |
| Diabetes | 1 | (100.0) | 0 | (0.0) | 0 | (0.0) | 0 | (0.0) |
| School absence due sickness | 1 | (100.0) | 0 | (0.0) | 0 | (0.0) | 0 | (0.0) |
| Not specified | 1 | (50.0) | 1 | (50.0) | 0 | (0.0) | 0 | (0.0) |

Note: ^*^Categories are not mutually exclusive.

**Supplementary Figure 1. Number of studies included per year.**


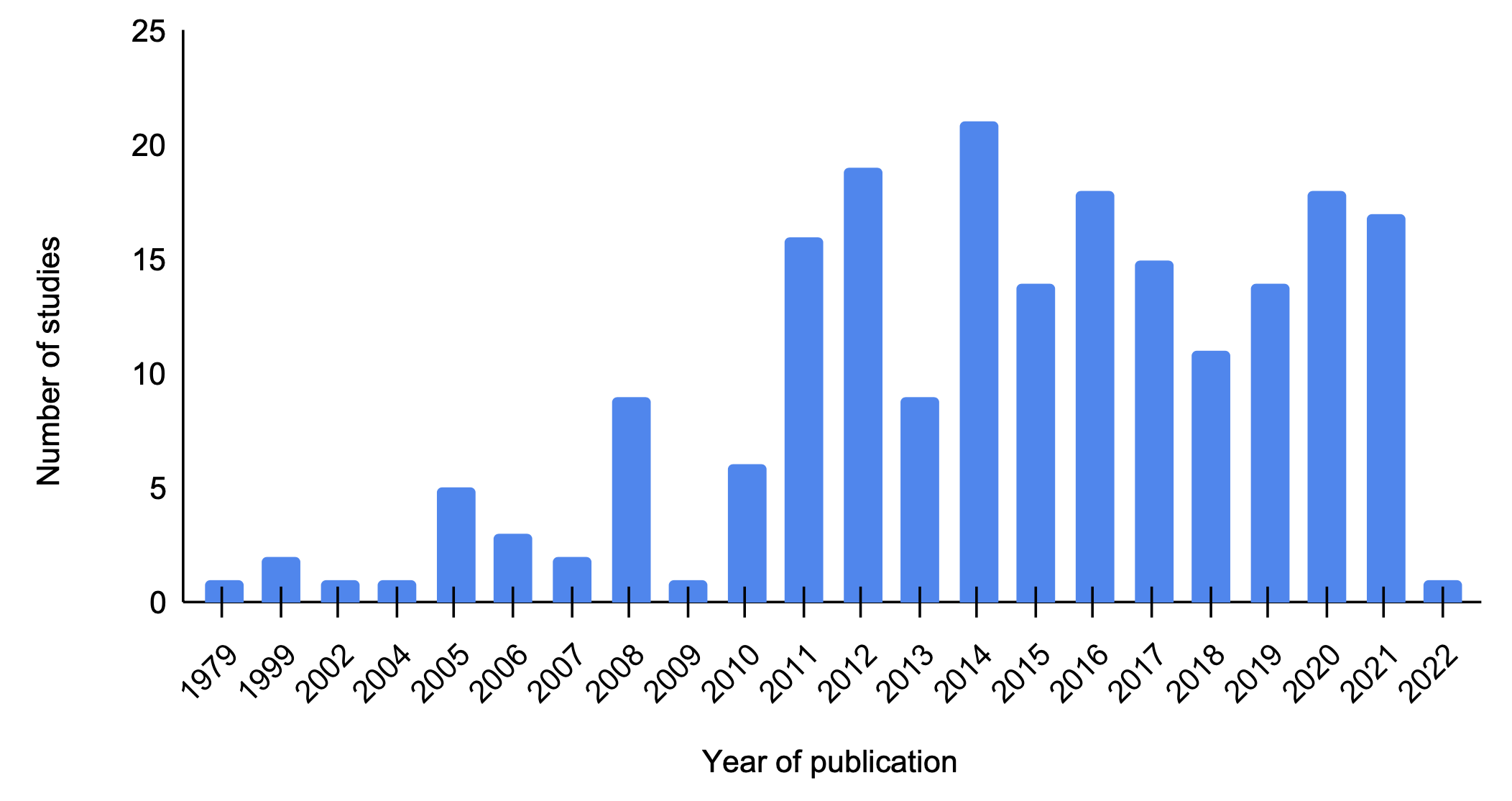


**Supplementary Figure 2.**


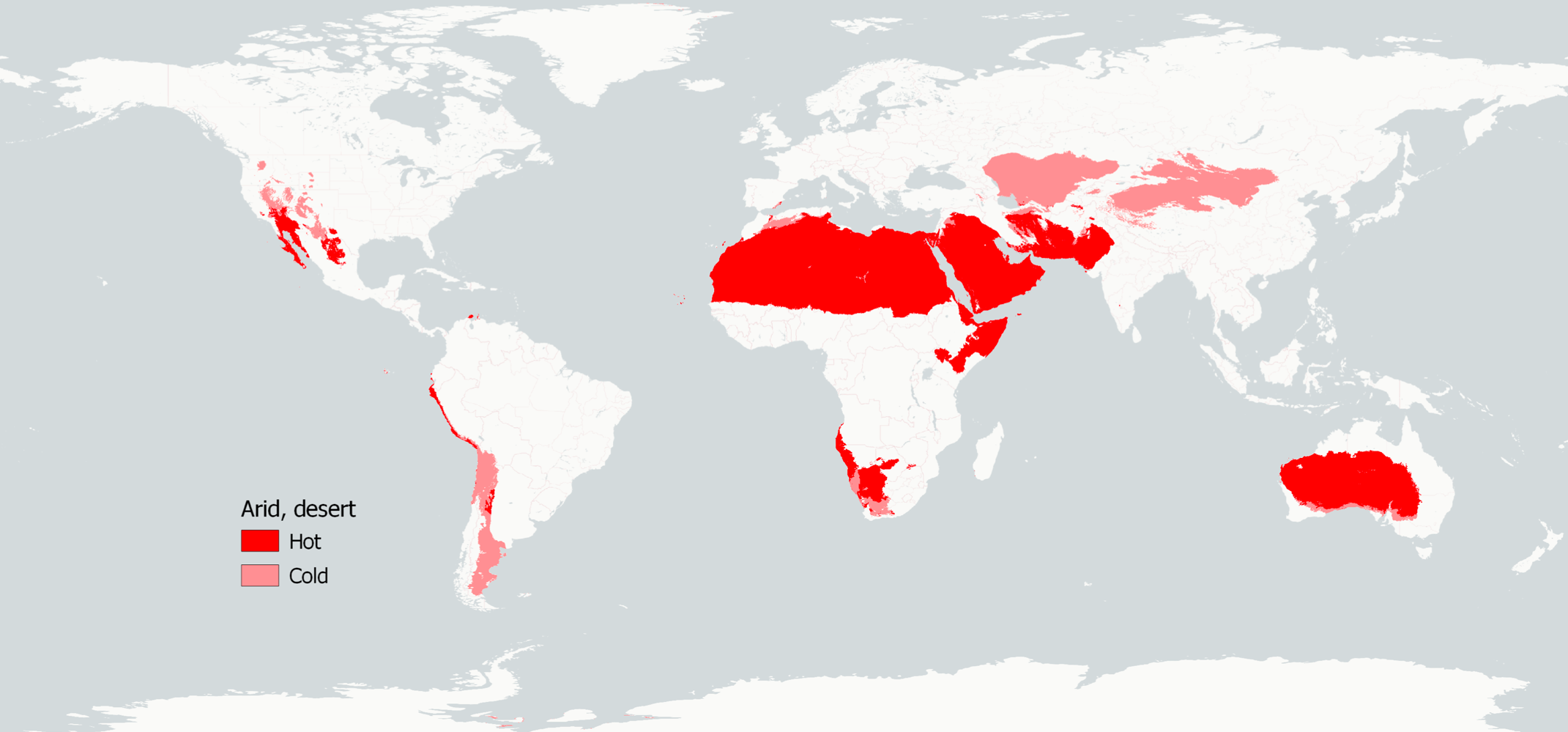


**List of studies included in the scoping review.**

Achakulwisut, P., L. J. Mickley and S. C. Anenberg (2018). "Drought-sensitivity of fine dust in the US Southwest: Implications for air quality and public health under future climate change." ENVIRONMENTAL RESEARCH LETTERS **13**(5).

Achilleos, S., E. Al-Ozairi, B. Alahmad, E. Garshick, A. M. Neophytou, W. Bouhamra, M. F. Yassin and P. Koutrakis (2019). "Acute effects of air pollution on mortality: A 17-year analysis in Kuwait." Environ Int **126**: 476-483.

Aghababaeian, H., M. Dastoorpoor, A. Ghasemi, M. Kiarsi, N. Khanjani and L. A. Ahvazi (2019). "Cardiovascular and respiratory emergency dispatch due to short-term exposure to ambient PM10 in Dezful, Iran." JOURNAL OF CARDIOVASCULAR AND THORACIC RESEARCH **11**(4): 264-271.

Aghababaeian, H., A. Ostadtaghizadeh, A. Ardalan, A. Asgary, M. Akbary, M. S. Yekaninejad, R. Sharafkhani and C. Stephens (2021). "Effect of Dust Storms on Non-Accidental, Cardiovascular, and Respiratory Mortality: A Case of Dezful City in Iran." ENVIRONMENTAL HEALTH INSIGHTS **15**.

Agier, L., A. Deroubaix, N. Martiny, P. Yaka, A. Djibo and H. Broutin (2013). "Seasonality of meningitis in Africa and climate forcing: aerosols stand out." JOURNAL OF THE ROYAL SOCIETY INTERFACE **10**(79).

Aili, A. S. J. and N. T. K. Oanh (2015). "Effects of dust storm on public health in desert fringe area: Case study of northeast edge of Taklimakan Desert, China." ATMOSPHERIC POLLUTION RESEARCH **6**(5): 805-814.

Akpinar-Elci, M., F. E. Martin, J. G. Behr and R. Diaz (2015). "Saharan dust, climate variability, and asthma in Grenada, the Caribbean." Int J Biometeorol **59**(11): 1667-1671.

Al, B., M. Bogan, S. Zengin, M. Sabak, S. Kul, M. M. Oktay, H. Bayram and E. Vuruskan (2018). "Effects of Dust Storms and Climatological Factors on Mortality and Morbidity of Cardiovascular Diseases Admitted to ED." Emerg Med Int **2018**: 3758506.

Al-Dousari, A. M., M. I. Ibrahim, N. Al-Dousari, M. Ahmed and S. Al-Awadhi (2018). "Pollen in aeolian dust with relation to allergy and asthma in Kuwait." AEROBIOLOGIA **34**(3): 325-336.

Al-Hemoud, A., A. Al-Dousari, A. Al-Shatti, A. Al-Khayat, W. Behbehani and M. Malak (2018). "Health Impact Assessment Associated with Exposure to PM10 and Dust Storms in Kuwait." ATMOSPHERE **9**(1).

Al-Rifaia, N., A. Elmi and A. R. Khan (2011). "Exploring possible seasonal trends in mortality rate in relation to age, gender and environmental conditions in Kuwait: A preliminary analysis." Journal of Engineering Research.

Al-Taiar, A. and L. Thalib (2014). "Short-term effect of dust storms on the risk of mortality due to respiratory, cardiovascular and all-causes in Kuwait." Int J Biometeorol **58**(1): 69-77.

Alangari, A. A., M. Riaz, M. O. Mahjoub, N. Malhis, S. Al-Tamimi and A. Al-Modaihsh (2015). "The effect of sand storms on acute asthma in Riyadh, Saudi Arabia." Ann Thorac Med **10**(1): 29-33.

Alessandrini, E. R., M. Stafoggia, A. Faustini, G. P. Gobbi and F. Forastiere (2013). "Saharan dust and the association between particulate matter and daily hospitalisations in Rome, Italy." Occup Environ Med **70**(6): 432-434.

Almasi, A., S. Bakhshi, M. Pirsaheb, S. A. Mousavi, M. Rezaei, E. Saleh and K. Sharafi (2016). "EFFECTS OF AIR POLLUTION CAUSED BY PARTICULATE MATTER (PM10) ON OURISMINDUSTRY AND ROADACCIDENTS-CASE STUDY: KERMANSHAH, IRAN (2008-2013)." ACTA MEDICA MEDITERRANEA **32**: 1951-1954.

Altindag, D. T., D. Baek and N. Mocan (2017). "Chinese Yellow Dust and Korean infant health." Soc Sci Med **186**: 78-86.

Aminharati, F., M. M. S. Dallal, M. H. Ehrampoush, A. Dehghani-Tafti, M. Yaseri, M. Memariani and Z. Rajabi (2018). "The effect of environmental parameters on the incidence of Shigella outbreaks in Yazd province, Iran." WATER SCIENCE AND TECHNOLOGY-WATER SUPPLY **18**(4): 1388-1395.

Aminharati, F., M. H. Ehrampoush, M. M. Soltan Dallal, M. Yaseri, A. A. Dehghani Tafti and Z. Rajabi (2019). "Citrobacter freundii Foodborne Disease Outbreaks Related to Environmental Conditions in Yazd Province, Iran." IRANIAN JOURNAL OF PUBLIC HEALTH **48**(6): 1099-1105.

Augusto, S., N. Ratola, P. Tarín-Carrasco, P. Jiménez-Guerrero, M. Turco, M. Schuhmacher, S. Costa, J. P. Teixeira and C. Costa (2020). "Population exposure to particulate-matter and related mortality due to the Portuguese wildfires in October 2017 driven by storm Ophelia." Environ Int **144**: 106056.

Bachwenkizi, J., C. Liu, X. Meng, L. N. Zhang, W. D. Wang, A. van Donkelaar, R. V. Martin, M. S. Hammer, R. J. Chen and H. D. Kan (2021). "Fine particulate matter constituents and infant mortality in Africa: A multicountry study." ENVIRONMENT INTERNATIONAL **156**.

Baltaci, H., H. Arslan and B. O. Akkoyunlu (2022). "High PM10 source regions and their influence on respiratory diseases in Canakkale, Turkey." INTERNATIONAL JOURNAL OF ENVIRONMENTAL SCIENCE AND TECHNOLOGY **19**(2): 797-806.

Barnett, A. G., J. F. Fraser and L. Munck (2012). "The effects of the 2009 dust storm on emergency admissions to a hospital in Brisbane, Australia." INTERNATIONAL JOURNAL OF BIOMETEOROLOGY **56**(4): 719-726.

Bell, M. L., J. K. Levy and Z. Lin (2008). "The effect of sandstorms and air pollution on cause-specific hospital admissions in Taipei, Taiwan." OCCUPATIONAL AND ENVIRONMENTAL MEDICINE **65**(2): 104-111.

Bennett, C. M., I. G. McKendry, S. Kelly, K. Denike and T. Koch (2006). "Impact of the 1998 Gobi dust event on hospital admissions in the Lower Fraser Valley, British Columbia." Sci Total Environ **366**(2): 918-925.

Bhattachan, A., G. S. Okin, J. Zhang, S. Vimal and D. P. Lettenmaier (2019). "Characterizing the Role of Wind and Dust in Traffic Accidents in California." GeoHealth.

Bogan, M., B. Al, S. Kul, S. Zengin, M. Oktay, M. Sabak, H. Gümüşboğa and H. Bayram (2021). "The effects of desert dust storms, air pollution, and temperature on morbidity due to spontaneous abortions and toxemia of pregnancy: 5-year analysis." Int J Biometeorol **65**(10): 1733-1739.

Byun, G., H. Kim, Y. Choi and J. T. Lee (2019). "The difference in effect of ambient particles on mortality between days with and without yellow dust events: Using a larger dataset in Seoul, Korea from 1998 to 2015." Sci Total Environ **691**: 819-826.

Cadelis, G., R. Tourres and J. Molinie (2014). "Short-term effects of the particulate pollutants contained in Saharan dust on the visits of children to the emergency department due to asthmatic conditions in Guadeloupe (French Archipelago of the Caribbean)." PLoS One **9**(3): e91136.

Chan, C. C., K. J. Chuang, W. J. Chen, W. T. Chang, C. T. Lee and C. M. Peng (2008). "Increasing cardiopulmonary emergency visits by long-range transported Asian dust storms in Taiwan." Environ Res **106**(3): 393-400.

Chan, C. C. and H. C. Ng (2011). "A case-crossover analysis of Asian dust storms and mortality in the downwind areas using 14-year data in Taipei." Sci Total Environ **410**: 47-52.

Chan, Y. S., J. C. Y. Teng, T. C. Liu and Y. I. Peng (2018). "Asian dust storms and diabetes hospitalization: a nationwide population-based study." AIR QUALITY ATMOSPHERE AND HEALTH **11**(10): 1243-1250.

Chang, C. C., I. M. Lee, S. S. Tsai and C. Y. Yang (2006). "Correlation of Asian dust storm events with daily clinic visits for allergic rhinitis in Taipei, Taiwan." J Toxicol Environ Health A **69**(3): 229-235.

Chen, C. S., Y. S. Chan and T. C. Liu (2021). "Tracheitis hospital admissions are associated with Asia dust storm." INTERNATIONAL JOURNAL OF ENVIRONMENTAL HEALTH RESEARCH.

Chen, Y. S., P. C. Sheen, E. R. Chen, Y. K. Liu, T. N. Wu and C. Y. Yang (2004). "Effects of Asian dust storm events on daily mortality in Taipei, Taiwan." Environ Res **95**(2): 151-155.

Chen, Y. S. and C. Y. Yang (2005). "Effects of Asian dust storm events on daily hospital admissions for cardiovascular disease in Taipei, Taiwan." J Toxicol Environ Health A **68**(17): 1457-1464.

Cheng, M. F., S. C. Ho, H. F. Chiu, T. N. Wu, P. S. Chen and C. Y. Yang (2008). "Consequences of exposure to Asian dust storm events on daily pneumonia hospital admissions in Taipei, Taiwan." J Toxicol Environ Health A **71**(19): 1295-1299.

Chien, L. C., Y. J. Lien, C. H. Yang and H. L. Yu (2014). "Acute increase of children's conjunctivitis clinic visits by Asian dust storms exposure - a spatiotemporal study in Taipei, Taiwan." PLoS One **9**(10): e109175.

Chien, L. C., C. H. Yang and H. L. Yu (2012). "Estimated effects of Asian dust storms on spatiotemporal distributions of clinic visits for respiratory diseases in Taipei children (Taiwan)." Environ Health Perspect **120**(8): 1215-1220.

Chiu, H. F., M. M. Tiao, S. C. Ho, H. W. Kuo, T. N. Wu and C. Y. Yang (2008). "Effects of Asian dust storm events on hospital admissions for chronic obstructive pulmonary disease in Taipei, Taiwan." Inhal Toxicol **20**(9): 777-781.

Comrie, A. C. (2021). "No Consistent Link Between Dust Storms and Valley Fever (Coccidioidomycosis)." Geohealth **5**(12): e2021GH000504.

Crooks, J. L., W. E. Cascio, M. S. Percy, J. Reyes, L. M. Neas and E. D. Hilborn (2016). "The Association between Dust Storms and Daily Non-Accidental Mortality in the United States, 1993-2005." ENVIRONMENTAL HEALTH PERSPECTIVES **124**(11): 1735-1743.

Dadvand, P., X. Basagaña, F. Figueras, E. Amoly, A. Tobias, A. de Nazelle, X. Querol, J. Sunyer and M. J. Nieuwenhuijsen (2011). "Saharan dust episodes and pregnancy." J Environ Monit **13**(11): 3222-3228.

Dallal, M. M. S., M. H. Ehrampoush, F. Aminharati, A. A. D. Tafti, M. Yaseri and M. Memariani (2020). "Associations between climatic parameters and the human salmonellosis in Yazd province, Iran." ENVIRONMENTAL RESEARCH **187**.

Dastoorpoor, M., E. Idani, G. Goudarzi and N. Khanjani (2018). "Acute effects of air pollution on spontaneous abortion, premature delivery, and stillbirth in Ahvaz, Iran: a time-series study." ENVIRONMENTAL SCIENCE AND POLLUTION RESEARCH **25**(6): 5447-5458.

De Longueville, F., Y. Hountondji, P. Ozer and S. Henry (2014). "The Air Quality in African Rural Environments. Preliminary Implications for Health: The Case of Respiratory Disease in the Northern Benin." WATER AIR AND SOIL POLLUTION **225**(11).

Desalu, O. O., A. O. Adeoti, O. B. Ojuawo, A. O. Aladesanmi, M. S. Oguntoye, O. J. Afolayan, M. O. Bojuwoye and A. E. Fawibe (2021). "Urban-Rural Differences in the Epidemiology of Asthma and Allergies in Nigeria: A Population-Based Study." JOURNAL OF ASTHMA AND ALLERGY **14**: 1389-1397.

Díaz, J., C. Linares, R. Carmona, A. Russo, C. Ortiz, P. Salvador and R. M. Trigo (2017). "Saharan dust intrusions in Spain: Health impacts and associated synoptic conditions." Environ Res **156**: 455-467.

Díaz, J., A. Tobías and C. Linares (2012). "Saharan dust and association between particulate matter and case-specific mortality: a case-crossover analysis in Madrid (Spain)." Environ Health **11**: 11.

Diokhane, A. M., G. S. Jenkins, N. Manga, M. S. Drame and B. Mbodji (2016). "Linkages between observed, modeled Saharan dust loading and meningitis in Senegal during 2012 and 2013." Int J Biometeorol **60**(4): 557-575.

Dogan, T. R., A. C. Saydam, M. I. Yesilnacar and M. Gencer (2010). "In-cloud alteration of desert-dust matrix and its possible impact on health: a test in southeastern Anatolia, Turkey." EUROPEAN JOURNAL OF MINERALOGY **22**(5): 659-664.

Domínguez-Rodríguez, A., N. Báez-Ferrer, P. Abreu-González, S. Rodríguez, R. Díaz, P. Avanzas and D. Hernández-Vaquero (2021). "Impact of Desert Dust Events on the Cardiovascular Disease: A Systematic Review and Meta-Analysis." J Clin Med **10**(4).

Dominguez-Rodriguez, A., N. Baez-Ferrer, S. Rodríguez, P. Avanzas, P. Abreu-Gonzalez, E. Terradellas, E. Cuevas, S. Basart and E. Werner (2020). "Saharan Dust Events in the Dust Belt -Canary Islands- and the Observed Association with in-Hospital Mortality of Patients with Heart Failure." J Clin Med **9**(2).

Dominguez-Rodriguez, A., S. Rodríguez, N. Baez-Ferrer, P. Abreu-Gonzalez, J. Abreu-Gonzalez, P. Avanzas, M. Carnero, C. Moris, J. López-Darias and D. Hernández-Vaquero (2020). "Impact of Saharan dust exposure on airway inflammation in patients with ischemic heart disease." Transl Res **224**: 16-25.

Ebenstein, A., E. Frank and Y. Reingewertz (2015). "Particulate Matter Concentrations, Sandstorms and Respiratory Hospital Admissions in Israel." ISRAEL MEDICAL ASSOCIATION JOURNAL **17**(10): 628-632.

Ebrahimi, S. J., L. Ebrahimzadeh, A. Eslami and F. Bidarpoor (2014). "Effects of dust storm events on emergency admissions for cardiovascular and respiratory diseases in Sanandaj, Iran." J Environ Health Sci Eng **12**: 110.

Garcia-Pando, C. P., M. C. Stanton, P. J. Diggle, S. Trzaska, R. L. Miller, J. P. Perlwitz, J. M. Baldasano, E. Cuevas, P. Ceccato, P. Yaka and M. C. Thomson (2014). "Soil Dust Aerosols and Wind as Predictors of Seasonal Meningitis Incidence in Niger." ENVIRONMENTAL HEALTH PERSPECTIVES **122**(7): 679-686.

Geravandi, S., P. Sicard, Y. O. Khaniabadi, A. De Marco, A. Ghomeishi, G. Goudarzi, M. Mahboubi, A. R. Yari, S. Dobaradaran, G. Hassani, M. J. Mohammadi and S. Sadeghi (2017). "A comparative study of hospital admissions for respiratory diseases during normal and dusty days in Iran." Environ Sci Pollut Res Int **24**(22): 18152-18159.

Gheybi, M. K., A. Movahed, R. Dehdari, S. Amiri, H. A. Khazaei, M. Gooya, F. Dehbashi, A. Fatemi, N. Sovid, G. Hajiani, R. Tahmasebi, S. Dobaradaran, M. Assadi and S. Farrokhi (2014). "Dusty Air Pollution is Associated with an Increased Risk of Allergic Diseases in Southwestern Part of Iran." IRANIAN JOURNAL OF ALLERGY ASTHMA AND IMMUNOLOGY **13**(6): 404-411.

Grineski, S. E., J. G. Staniswalis, P. Bulathsinhala, Y. Peng and T. E. Gill (2011). "Hospital admissions for asthma and acute bronchitis in El Paso, Texas: do age, sex, and insurance status modify the effects of dust and low wind events?" Environ Res **111**(8): 1148-1155.

Gupta, P., S. Singh, S. Kumar, M. Choudhary and V. Singh (2012). "Effect of Dust Aerosol in Patients with Asthma." JOURNAL OF ASTHMA **49**(2): 134-138.

Gutierrez, M. P., P. Zuidema, M. Mirsaeidi, M. Campos and N. Kumar (2020). "Association between African Dust Transport and Acute Exacerbations of COPD in Miami." J Clin Med **9**(8).

Gyan, K., W. Henry, S. Lacaille, A. Laloo, C. Lamsee-Ebanks, S. McKay, R. M. Antoine and M. A. Monteil (2005). "African dust clouds are associated with increased paediatric asthma accident and emergency admissions on the Caribbean island of Trinidad." Int J Biometeorol **49**(6): 371-376.

Hasunuma, H., A. Takeuchi, R. Ono, Y. Amimoto, Y. H. Hwang, I. Uno, A. Shimizu, Y. Nishiwaki, M. Hashizume, D. J. Askew and H. Odajima (2021). "Effect of Asian dust on respiratory symptoms among children with and without asthma, and their sensitivity." Sci Total Environ **753**: 141585.

Hayilu, D., K. Legesse, N. Lakachew and M. Asferaw (2016). "Prevalence and associated factors of vernal keratoconjunctivitis among children in Gondar city, Northwest Ethiopia." BMC OPHTHALMOLOGY **16**.

Heft-Neal, S., J. Burney, E. Bendavid, K. K. Voss and M. Burke (2020). "Dust pollution from the Sahara and African infant mortality." NATURE SUSTAINABILITY **3**(10): 863-+.

Herrera-Molina, E., T. E. Gill, G. Ibarra-Mejia and S. Jeon (2021). "Associations between Dust Exposure and Hospitalizations in El Paso, Texas, USA." ATMOSPHERE **12**(11).

Higashi, T., Y. Kambayashi, N. Ohkura, M. Fujimura, S. Nakai, Y. Honda, K. Saijoh, K. Hayakawa, F. Kobayashi, Y. Michigami, A. E. Olando, Y. Hitomi and H. Nakamura (2014). "Effects of Asian dust on daily cough occurrence in patients with chronic cough: A panel study." ATMOSPHERIC ENVIRONMENT **92**: 506-513.

Higashi, T., Y. Kambayashi, N. Ohkura, M. Fujimura, S. Nakanishi, T. Yoshizaki, K. Saijoh, K. Hayakawa, F. Kobayashi, Y. Michigami, Y. Hitomi and H. Nakamura (2014). "Exacerbation of daily cough and allergic symptoms in adult patients with chronic cough by Asian dust: A hospital-based study in Kanazawa." ATMOSPHERIC ENVIRONMENT **97**: 537-543.

Ho, H. C., M. S. Wong, L. Yang, T. C. Chan and M. Bilal (2018). "Influences of socioeconomic vulnerability and intra-urban air pollution exposure on short-term mortality during extreme dust events." Environ Pollut **235**: 155-162.

Hong, Y. C., X. C. Pan, S. Y. Kim, K. Park, E. J. Park, X. Jin, S. M. Yi, Y. H. Kim, C. H. Park, S. Song and H. Kim (2010). "Asian Dust Storm and pulmonary function of school children in Seoul." Sci Total Environ **408**(4): 754-759.

Ishii, M., T. Seki, K. Kaikita, K. Sakamoto, M. Nakai, Y. Sumita, K. Nishimura, Y. Miyamoto, T. Noguchi, S. Yasuda, H. Tsutsui, I. Komuro, Y. Saito, H. Ogawa, K. Tsujita and K. Kawakami (2020). "Short-term exposure to desert dust and the risk of acute myocardial infarction in Japan: a time-stratified case-crossover study." Eur J Epidemiol **35**(5): 455-464.

Ishii, M., T. Seki, K. Sakamoto, K. Kaikita, Y. Miyamoto, K. Tsujita, I. Masuda and K. Kawakami (2020). "Association of short term exposure to Asian dust with increased blood pressure." Sci Rep **10**(1): 17630.

Itazawa, T., K. T. Kanatani, K. Hamazaki, H. Inadera, A. Tsuchida, T. Tanaka, T. Nakayama, T. Go, K. Onishi, Y. Kurozawa and Y. Adachi (2020). "The impact of exposure to desert dust on infants' symptoms and countermeasures to reduce the effects." Allergy **75**(6): 1435-1445.

Jiménez, E., C. Linares, D. Martínez and J. Díaz (2010). "Role of Saharan dust in the relationship between particulate matter and short-term daily mortality among the elderly in Madrid (Spain)." Sci Total Environ **408**(23): 5729-5736.

Johnston, F., I. Hanigan, S. Henderson, G. Morgan and D. Bowman (2011). "Extreme air pollution events from bushfires and dust storms and their association with mortality in Sydney, Australia 1994-2007." Environ Res **111**(6): 811-816.

Jones, B. A. (2020). "After the Dust Settles: The Infant Health Impacts of Dust Storms." JOURNAL OF THE ASSOCIATION OF ENVIRONMENTAL AND RESOURCE ECONOMISTS **7**(6): 1005-1032.

Kamouchi, M., K. Ueda, T. Ago, H. Nitta and T. Kitazono (2012). "Relationship between asian dust and ischemic stroke: a time-stratified case-crossover study." Stroke **43**(11): 3085-3087.

Kanatani, K. T., K. Hamazaki, H. Inadera, N. Sugimoto, A. Shimizu, H. Noma, K. Onishi, Y. Takahashi, T. Itazawa, M. Egawa, K. Sato, T. Go, I. Ito, Y. Kurozawa, I. Konishi, Y. Adachi and T. Nakayama (2016). "Effect of desert dust exposure on allergic symptoms: A natural experiment in Japan." Ann Allergy Asthma Immunol **116**(5): 425-430.e427.

Kanatani, K. T., I. Ito, W. K. Al-Delaimy, Y. Adachi, W. C. Mathews and J. W. Ramsdell (2010). "Desert dust exposure is associated with increased risk of asthma hospitalization in children." Am J Respir Crit Care Med **182**(12): 1475-1481.

Kang, J. H., J. J. Keller, C. S. Chen and H. C. Lin (2012). "Asian dust storm events are associated with an acute increase in pneumonia hospitalization." Ann Epidemiol **22**(4): 257-263.

Kang, J. H., T. C. Liu, J. Keller and H. C. Lin (2013). "Asian dust storm events are associated with an acute increase in stroke hospitalisation." J Epidemiol Community Health **67**(2): 125-131.

Karimi, S. M., H. Pouran, M. Majbouri, M. Moradi-Lakeh and H. Hakimian (2020). "Saharan sand and dust storms and neonatal mortality: Evidence from Burkina Faso." Sci Total Environ **729**: 139053.

Kashima, S., T. Yorifuji, S. Bae, Y. Honda, Y. H. Lim and Y. C. Hong (2016). "Asian dust effect on cause-specific mortality in five cities across South Korea and Japan." ATMOSPHERIC ENVIRONMENT **128**: 20-27.

Kashima, S., T. Yorifuji and E. Suzuki (2014). "Asian dust and daily emergency ambulance calls among elderly people in Japan: an analysis of its double role as a direct cause and as an effect modifier." J Occup Environ Med **56**(12): 1277-1283.

Kashima, S., T. Yorifuji and E. Suzuki (2017). "Are People With a History of Disease More Susceptible to a Short-term Exposure to Asian Dust?: A Case-Crossover Study Among the Elderly in Japan." Epidemiology **28**: S60-s66.

Kashima, S., T. Yorifuji, T. Tsuda and A. Eboshida (2012). "Asian dust and daily all-cause or cause-specific mortality in western Japan." Occup Environ Med **69**(12): 908-915.

Kim, H. S., D. S. Kim, H. Kim and S. M. Yi (2012). "Relationship between mortality and fine particles during Asian dust, smog-Asian dust, and smog days in Korea." Int J Environ Health Res 22(6): 518-530.

Ko, R., M. Hayashi, H. Hayashi, K. Hayashi, H. Kato, Y. Kurata, Y. Fuchino, T. Nakamichi, H. Migita, H. Yano, T. Sakata and E. Uchio (2016). "Correlation between acute conjunctivitis and Asian dust on ocular surfaces." J Toxicol Environ Health A 79(8): 367-375.

Kojima, S., T. Michikawa, K. Ueda, T. Sakamoto, K. Matsui, T. Kojima, K. Tsujita, H. Ogawa, H. Nitta and A. Takami (2017). "Asian dust exposure triggers acute myocardial infarction." Eur Heart J 38(43): 3202-3208.

Kurai, J., M. Watanabe, H. Noma, K. Iwata, J. Taniguchi, H. Sano, Y. Tohda and E. Shimizu (2017). "Estimation of the effects of heavy Asian dust on respiratory function by definition type." Genes Environ 39: 25.

Kwon, H. J., S. H. Cho, Y. Chun, F. Lagarde and G. Pershagen (2002). "Effects of the Asian dust events on daily mortality in Seoul, Korea." Environ Res 90(1): 1-5.

Lai, L. W. and W. L. Cheng (2008). "The impact of air quality on respiratory admissions during Asian dust storm periods." Int J Environ Health Res 18(6): 429-450.

Lee, H., Y. Honda, Y. H. Lim, Y. L. Guo, M. Hashizume and H. Kim (2014). "Effect of Asian dust storms on mortality in three Asian cities." ATMOSPHERIC ENVIRONMENT 89: 309-317.

Lee, H., J. Jung, W. Myung, J. H. Baek, J. M. Kang, D. K. Kim and H. Kim (2019). "Association between dust storm occurrence and risk of suicide: Case-crossover analysis of the Korean national death database." Environ Int 133: 105146.

Lee, H., H. Kim, Y. Honda, Y. H. Lim and S. Yi (2013). "Effect of Asian dust storms on daily mortality in seven metropolitan cities of Korea." ATMOSPHERIC ENVIRONMENT 79: 510-517.

Lee, J. T., J. Y. Son and Y. S. Cho (2007). "A comparison of mortality related to urban air particles between periods with Asian dust days and without Asian dust days in Seoul, Korea, 2000-2004." Environ Res 105(3): 409-413.

Lee, J. W. and K. K. Lee (2014). "Effects of Asian dust events on daily asthma patients in Seoul, Korea." METEOROLOGICAL APPLICATIONS 21(2): 202-209.

Lee, S., W. Lee, E. Lee, M. H. Jeong, S. W. Rha, C. J. Kim, S. C. Chae, H. S. Kim, H. C. Gwon and H. Kim (2021). "Effects of Asian dust-derived particulate matter on ST-elevation myocardial infarction: retrospective, time series study." BMC Public Health **21**(1): 68.

Li, Z. H., L. Chen, M. Q. Li and J. Cohen (2018). "Prenatal exposure to sand and dust storms and children's cognitive function in China: a quasi-experimental study." LANCET PLANETARY HEALTH **2**(5): E214-E222.

Lien, W. H., P. O. Owili, M. A. Muga and T. H. Lin (2019). "Ambient Particulate Matter Exposure and Under-Five and Maternal Deaths in Asia." INTERNATIONAL JOURNAL OF ENVIRONMENTAL RESEARCH AND PUBLIC HEALTH **16**(20).

Lin, Y. K., C. F. Chen, H. C. Yeh and Y. C. Wang (2016). "Emergency room visits associated with particulate concentration and Asian dust storms in metropolitan Taipei." J Expo Sci Environ Epidemiol **26**(2): 189-196.

Linares, C., D. Culqui, F. Belda, J. A. López-Bueno, Y. Luna, G. Sánchez-Martínez, B. Hervella and J. Díaz (2021). "Impact of environmental factors and Sahara dust intrusions on incidence and severity of COVID-19 disease in Spain. Effect in the first and second pandemic waves." Environ Sci Pollut Res Int **28**(37): 51948-51960.

Liu, S. T., C. Y. Liao, C. Y. Kuo and H. W. Kuo (2017). "The Effects of PM(2.5) from Asian Dust Storms on Emergency Room Visits for Cardiovascular and Respiratory Diseases." Int J Environ Res Public Health **14**(4).

López-Villarrubia, E., O. Costa Estirado, C. Íñiguez Hernández and F. Ballester Díez (2020). "Do Saharan Dust Days Carry a Risk of Hospitalization From Respiratory Diseases for Citizens of the Canary Islands (Spain)?" Arch Bronconeumol (Engl Ed).

Lorentzou, C., G. Kouvarakis, G. V. Kozyrakis, N. A. Kampanis, I. Trahanatzi, O. Fraidakis, N. Tzanakis, M. Kanakidou, P. Agouridakis and G. Notas (2019). "Extreme desert dust storms and COPD morbidity on the island of Crete." Int J Chron Obstruct Pulmon Dis **14**: 1763-1768.

Ma, Y., H. Zhang, Y. Zhao, J. Zhou, S. Yang, X. Zheng and S. Wang (2017). "Short-term effects of air pollution on daily hospital admissions for cardiovascular diseases in western China." Environ Sci Pollut Res Int **24**(16): 14071-14079.

Ma, Y. X., B. S. Xiao, C. Liu, Y. X. Zhao and X. D. Zheng (2016). "Association between Ambient Air Pollution and Emergency Room Visits for Respiratory Diseases in Spring Dust Storm Season in Lanzhou, China." INTERNATIONAL JOURNAL OF ENVIRONMENTAL RESEARCH AND PUBLIC HEALTH **13**(6).

Ma, Y. X., J. D. Zhou, S. X. Yang, Y. X. Zhao and X. D. Zheng (2017). "Assessment for the impact of dust events on measles incidence in western China." ATMOSPHERIC ENVIRONMENT **157**: 1-9.

Majbauddin, A., K. Onishi, S. Otani, Y. Kurosaki and Y. Kurozawa (2016). "Association between Asian Dust-Borne Air Pollutants and Daily Symptoms on Healthy Subjects: A Web-Based Pilot Study in Yonago, Japan." J Environ Public Health **2016**: 8280423.

Mallone, S., M. Stafoggia, A. Faustini, G. P. Gobbi, A. Marconi and F. Forastiere (2011). "Saharan dust and associations between particulate matter and daily mortality in Rome, Italy." Environ Health Perspect **119**(10): 1409-1414.

Martiny, N. and I. Chiapello (2013). "Assessments for the impact of mineral dust on the meningitis incidence in West Africa." ATMOSPHERIC ENVIRONMENT **70**: 245-253.

Matsukawa, R., T. Michikawa, K. Ueda, H. Nitta, T. Kawasaki, H. Tashiro, M. Mohri and Y. Yamamoto (2014). "Desert dust is a risk factor for the incidence of acute myocardial infarction in Western Japan." Circ Cardiovasc Qual Outcomes **7**(5): 743-748.

Menéndez, I., E. Derbyshire, T. Carrillo, E. Caballero, J. P. Engelbrecht, L. E. Romero, P. L. Mayer, F. Rodríguez de Castro and J. Mangas (2017). "Saharan dust and the impact on adult and elderly allergic patients: the effect of threshold values in the northern sector of Gran Canaria, Spain." Int J Environ Health Res **27**(2): 144-160.

Meng, Z. Q. and B. Lu (2007). "Dust events as a risk factor for daily hospitalization for respiratory and cardiovascular diseases in Minqin, China." ATMOSPHERIC ENVIRONMENT **41**(33): 7048-7058.

Meo, S. A., M. F. A. Al-Kheraiji, Z. F. AlFaraj, N. A. Alwehaibi and A. A. Aldereihim (2013). "Respiratory and general health complaints in subjects exposed to sandstorm at Riyadh, Saudi Arabia." PAKISTAN JOURNAL OF MEDICAL SCIENCES **29**(2): 642-646.

Meo, S. A., F. J. Almutairi, A. A. Abukhalaf, O. M. Alessa, T. Al-Khlaiwi and A. S. Meo (2021). "Sandstorm and its effect on particulate matter PM 2.5, carbon monoxide, nitrogen dioxide, ozone pollutants and SARS-CoV-2 cases and deaths." SCIENCE OF THE TOTAL ENVIRONMENT **795**.

Merrifield, A., S. Schindeler, B. Jalaludin and W. Smith (2013). "Health effects of the September 2009 dust storm in Sydney, Australia: did emergency department visits and hospital admissions increase?" ENVIRONMENTAL HEALTH **12**.

Michikawa, T., S. Yamazaki, A. Shimizu, H. Nitta, K. Kato, Y. Nishiwaki and S. Morokuma (2020). "Exposure to Asian dust within a few days of delivery is associated with placental abruption in Japan: a case-crossover study." Bjog **127**(3): 335-342.

Middleton, N., P. Yiallouros, S. Kleanthous, O. Kolokotroni, J. Schwartz, D. W. Dockery, P. Demokritou and P. Koutrakis (2008). "A 10-year time-series analysis of respiratory and cardiovascular morbidity in Nicosia, Cyprus: the effect of short-term changes in air pollution and dust storms." ENVIRONMENTAL HEALTH **7**.

Mimura, T., S. Yamagami, H. Fujishima, H. Noma, Y. Kamei, M. Goto, A. Kondo and M. Matsubara (2014). "Sensitization to Asian dust and allergic rhinoconjunctivitis." Environ Res **132**: 220-225.

Moreira, I., C. Linares, F. Follos, G. Sánchez-Martínez, J. M. Vellón and J. Díaz (2020). "Short-term effects of Saharan dust intrusions and biomass combustion on birth outcomes in Spain." Sci Total Environ **701**: 134755.

Mu, H., B. Battsetseg, T. Y. Ito, S. Otani, K. Onishi and Y. Kurozawa (2010). "Effects of Asian dust storm on health-related quality of life: A survey immediately after an Asian dust storm event in Mongolia." International Journal of Health Research.

Mu, H. S., B. Battsetseg, T. Y. Ito, S. Otani, K. Onishi and Y. Kurozawa (2011). "Health Effects of Dust Storms: Subjective Eye and Respiratory System Symptoms in Inhabitants in Mongolia." JOURNAL OF ENVIRONMENTAL HEALTH **73**(8): 18-20.

Nakamura, T., M. Hashizume, K. Ueda, T. Kubo, A. Shimizu, T. Okamura and Y. Nishiwaki (2015). "The relationship between Asian dust events and out-of-hospital cardiac arrests in Japan." J Epidemiol **25**(4): 289-296.

Nakamura, T., M. Hashizume, K. Ueda, A. Shimizu, A. Takeuchi, T. Kubo, K. Hashimoto, H. Moriuchi, H. Odajima, T. Kitajima, K. Tashiro, K. Tomimasu and Y. Nishiwaki (2016). "Asian Dust and Pediatric Emergency Department Visits Due to Bronchial Asthma and Respiratory Diseases in Nagasaki, Japan." J Epidemiol **26**(11): 593-601.

Nakamura, T., Y. Nishiwaki, K. Hashimoto, A. Takeuchi, T. Kitajima, K. Komori, K. Tashiro, H. Hasunuma, K. Ueda, A. Shimizu, H. Odajima, H. Moriuchi and M. Hashizume (2020). "Association between Asian dust exposure and respiratory function in children with bronchial asthma in Nagasaki Prefecture, Japan." Environ Health Prev Med **25**(1): 8.

Nakao, M., Y. Ishihara, C. H. Kim and I. G. Hyun (2018). "The Impact of Air Pollution, Including Asian Sand Dust, on Respiratory Symptoms and Health-related Quality of Life in Outpatients With Chronic Respiratory Disease in Korea: A Panel Study." J Prev Med Public Health **51**(3): 130-139.

Nakao, M., K. Yamauchi, S. Mitsuma, T. Odaira, H. Obata, Y. Chijimatsu and Y. Ishihara (2019). "Associations of Ambient Air Pollutant Concentrations With Respiratory Symptoms and Perceived Health Status in Japanese Adults With and Without Chronic Respiratory Diseases: A Panel Study." J Prev Med Public Health **52**(6): 416-426.

Nakao, M., K. Yamauchi, S. Mitsuma, H. Omori and Y. Ishihara (2019). "Relationships between perceived health status and ambient air quality parameters in healthy Japanese: a panel study." BMC PUBLIC HEALTH **19**.

Nastos, P. T., N. A. Kampanis, K. N. Giaouzaki and A. Matzarakis (2011). "Environmental impacts on human health during a Saharan dust episode at Crete Island, Greece." METEOROLOGISCHE ZEITSCHRIFT **20**(5): 517-529.

Neophytou, A. M., P. Yiallouros, B. A. Coull, S. Kleanthous, P. Pavlou, S. Pashiardis, D. W. Dockery, P. Koutrakis and F. Laden (2013). "Particulate matter concentrations during desert dust outbreaks and daily mortality in Nicosia, Cyprus." J Expo Sci Environ Epidemiol **23**(3): 275-280.

Ng, C. F. S., M. Hashizume, Y. Obase, M. Doi, K. Tamura, S. Tomari, T. Kawano, C. Fukushima, H. Matsuse, Y. Chung, Y. Kim, K. Kunimitsu, S. Kohno and H. Mukae (2019). "Associations of chemical composition and sources of PM(2.5) with lung function of severe asthmatic adults in a low air pollution environment of urban Nagasaki, Japan." Environ Pollut **252**: 599-606.

Onishi, K., Y. Kurosaki, S. Otani, A. Yoshida, N. Sugimoto and Y. Kurozawa (2012). "Atmospheric transport route determines components of Asian dust and health effects in Japan." ATMOSPHERIC ENVIRONMENT **49**: 94-102.

Onishi, K., S. Otani, A. Yoshida, H. Mu and Y. Kurozawa (2015). "Adverse health effects of Asian dust particles and heavy metals in Japan." Asia Pac J Public Health **27**(2): Np1719-1726.

Otani, S., K. Onishi, H. Mu, T. Hosoda, Y. Kurozawa and M. Ikeguchi (2014). "Associations between subjective symptoms and serum immunoglobulin E levels during Asian dust events." Int J Environ Res Public Health **11**(8): 7636-7641.

Otani, S., K. Onishi, H. Mu and Y. Kurozawa (2011). "The effect of Asian dust events on the daily symptoms in Yonago, Japan: a pilot study on healthy subjects." Arch Environ Occup Health **66**(1): 43-46.

Otani, S., K. Onishi, H. Mu, Y. Yokoyama, T. Hosoda, M. Okamoto and Y. Kurozawa (2012). "The relationship between skin symptoms and allergic reactions to Asian dust." Int J Environ Res Public Health **9**(12): 4606-4614.

Pandolfi, M., A. Tobias, A. Alastuey, J. Sunyer, J. Schwartz, J. Lorente, J. Pey and X. Querol (2014). "Effect of atmospheric mixing layer depth variations on urban air quality and daily mortality during Saharan dust outbreaks." Sci Total Environ **494**: 283-289.

Park, J., M. N. Lim, Y. Hong and W. J. Kim (2015). "The Influence of Asian Dust, Haze, Mist, and Fog on Hospital Visits for Airway Diseases." Tuberc Respir Dis (Seoul) **78**(4): 326-335.

Park, J. W., Y. H. Lim, S. Y. Kyung, C. H. An, S. P. Lee, S. H. Jeong and Y. S. Ju (2005). "Effects of ambient particulate matter on peak expiratory flow rates and respiratory symptoms of asthmatics during Asian dust periods in Korea." Respirology **10**(4): 470-476.

Park, Y. S., J. H. Kim, H. J. Jang, Y. H. Tae and D. H. Lim (2016). "The effect of Asian dust on asthma by socioeconomic status using national health insurance claims data in Korea." Inhal Toxicol **28**(1): 1-6.

Perez, L., A. Tobias, X. Querol, N. Künzli, J. Pey, A. Alastuey, M. Viana, N. Valero, M. González-Cabré and J. Sunyer (2008). "Coarse particles from Saharan dust and daily mortality." Epidemiology **19**(6): 800-807.

Perez, L., A. Tobías, X. Querol, J. Pey, A. Alastuey, J. Díaz and J. Sunyer (2012). "Saharan dust, particulate matter and cause-specific mortality: a case-crossover study in Barcelona (Spain)." Environ Int **48**: 150-155.

Prospero, J. M., E. Blades, R. Naidu, G. Mathison, H. Thani and M. C. Lavoie (2008). "Relationship between African dust carried in the Atlantic trade winds and surges in pediatric asthma attendances in the Caribbean." Int J Biometeorol **52**(8): 823-832.

Radmanesh, E., H. Maleki, G. Goudarzi, A. Zahedi, S. G. Kalkhajeh, P. K. Hopke, S. A. Mard and S. Olad (2019). "Cerebral ischemic attack, epilepsy and hospital admitted patients with types of headaches attributed to PM10 mass concentration in Abadan, Iran." AEOLIAN RESEARCH **41**.

Renzi, M., F. Forastiere, R. Calzolari, A. Cernigliaro, G. Madonia, P. Michelozzi, M. Davoli, S. Scondotto and M. Stafoggia (2018). "Short-term effects of desert and non-desert PM(10) on mortality in Sicily, Italy." Environ Int **120**: 472-479.

Reyes, M., J. Díaz, A. Tobias, J. C. Montero and C. Linares (2014). "Impact of Saharan dust particles on hospital admissions in Madrid (Spain)." Int J Environ Health Res **24**(1): 63-72.

Rublee, C. S., C. J. Sorensen, J. Lemery, T. J. Wade, E. A. Sams, E. D. Hilborn and J. L. Crooks (2020). "Associations Between Dust Storms and Intensive Care Unit Admissions in the United States, 2000-2015." GEOHEALTH **4**(8).

Rutherford, S., E. Clark, G. McTainsh, R. Simpson and C. Mitchell (1999). "Characteristics of rural dust events shown to impact on asthma severity in Brisbane, Australia." Int J Biometeorol **42**(4): 217-225.

Sadeghimoghaddam, A., H. Khankeh, M. Norozi, S. Fateh and M. Farrokhi (2021). "Effects of dust events and meteorological elements on stroke morbidity in northern Khuzestan, Iran." J Educ Health Promot **10**: 406.

Saers, J., L. Andersson, C. Janson and J. Sundh (2021). "Respiratory symptoms, lung function, and fraction of exhaled nitric oxide before and after assignment in a desert environment-a cohort study." RESPIRATORY MEDICINE **189**.

Sakata, S., S. Konishi, C. F. S. Ng, R. Kishikawa and C. Watanabe (2017). "Association of Asian Dust with daily medical consultations for pollinosis in Fukuoka City, Japan." Environ Health Prev Med **22**(1): 25.

Samoli, E., E. Kougea, P. Kassomenos, A. Analitis and K. Katsouyanni (2011). "Does the presence of desert dust modify the effect of PM10 on mortality in Athens, Greece?" Sci Total Environ **409**(11): 2049-2054.

Samoli, E., P. T. Nastos, A. G. Paliatsos, K. Katsouyanni and K. N. Priftis (2011). "Acute effects of air pollution on pediatric asthma exacerbation: evidence of association and effect modification." Environ Res **111**(3): 418-424.

Schwartz, J., G. Norris, T. Larson, L. Sheppard, C. Claiborn and J. Koenig (1999). "Episodes of high coarse particle concentrations are not associated with increased mortality." ENVIRONMENTAL HEALTH PERSPECTIVES **107**(5): 339-342.

Shahsavani, A., A. Tobías, X. Querol, M. Stafoggia, M. Abdolshahnejad, F. Mayvaneh, Y. Guo, M. Hadei, S. Saeed Hashemi, A. Khosravi, Z. Namvar, M. Yarahmadi and B. Emam (2020). "Short-term effects of particulate matter during desert and non-desert dust days on mortality in Iran." Environ Int **134**: 105299.

Silva, T., M. Fragoso, R. Almendra, J. Vasconcelos, A. Lopes and A. Faleh (2021). "North African dust intrusions and increased risk of respiratory diseases in Southern Portugal." Int J Biometeorol **65**(10): 1767-1780.

Soleimani, Z., A. D. Boloorani, R. Khalifeh, D. W. Griffin and A. Mesdaghinia (2019). "Short-term effects of ambient air pollution and cardiovascular events in Shiraz, Iran, 2009 to 2015." ENVIRONMENTAL SCIENCE AND POLLUTION RESEARCH **26**(7): 6359-6367.

Stafoggia, M., S. Zauli-Sajani, J. Pey, E. Samoli, E. Alessandrini, X. Basagaña, A. Cernigliaro, M. Chiusolo, M. Demaria, J. Díaz, A. Faustini, K. Katsouyanni, A. G. Kelessis, C. Linares, S. Marchesi, S. Medina, P. Pandolfi, N. Pérez, X. Querol, G. Randi, A. Ranzi, A. Tobias and F. Forastiere (2016). "Desert Dust Outbreaks in Southern Europe: Contribution to Daily PM₁₀ Concentrations and Short-Term Associations with Mortality and Hospital Admissions." Environ Health Perspect **124**(4): 413-419.

Tam, W. W. S., T. W. Wong and A. H. S. Wong (2012). "Effect of Dust Storm Events on Daily Emergency Admissions for Cardiovascular Diseases." CIRCULATION JOURNAL **76**(3): 655-660.

Tam, W. W. S., T. W. Wong, A. H. S. Wong and D. S. C. Hui (2012). "Effect of dust storm events on daily emergency admissions for respiratory diseases." RESPIROLOGY **17**(1): 143-148.

Tao, Y., X. Q. An, Z. B. Sun, Q. Hou and Y. Wang (2012). "Association between dust weather and number of admissions for patients with respiratory diseases in spring in Lanzhou." SCIENCE OF THE TOTAL ENVIRONMENT **423**: 8-11.

Teng, J. C., Y. S. Chan, Y. I. Peng and T. C. Liu (2016). "Influence of Asian dust storms on daily acute myocardial infarction hospital admissions." Public Health Nurs **33**(2): 118-128.

Thalib, L. and A. Al-Taiar (2012). "Dust storms and the risk of asthma admissions to hospitals in Kuwait." Sci Total Environ **433**: 347-351.

Tobías, A., J. A. Caylà, J. Pey, A. Alastuey and X. Querol (2011). "Are Saharan dust intrusions increasing the risk of meningococcal meningitis?" Int J Infect Dis **15**(7): e503.

Tobías, A., L. Pérez, J. Díaz, C. Linares, J. Pey, A. Alastruey and X. Querol (2011). "Short-term effects of particulate matter on total mortality during Saharan dust outbreaks: a case-crossover analysis in Madrid (Spain)." Sci Total Environ **412**: 386-389.

Tobías, A. and M. Stafoggia (2020). "Modeling Desert Dust Exposures in Epidemiologic Short-term Health Effects Studies." Epidemiology **31**(6): 788-795.

Tong, D. Q., J. X. L. Wang, T. E. Gill, H. Lei and B. Y. Wang (2017). "Intensified dust storm activity and Valley fever infection in the southwestern United States." GEOPHYSICAL RESEARCH LETTERS **44**(9): 4304-4312.

Trianti, S. M., E. Samoli, S. Rodopoulou, K. Katsouyanni, S. A. Papiris and A. Karakatsani (2017). "Desert dust outbreaks and respiratory morbidity in Athens, Greece." Environ Health **16**(1): 72.

Ueda, K., H. Nitta and H. Odajima (2010). "The effects of weather, air pollutants, and Asian dust on hospitalization for asthma in Fukuoka." Environ Health Prev Med **15**(6): 350-357.

Ueda, K., A. Shimizu, H. Nitta and K. Inoue (2012). "Long-range transported Asian Dust and emergency ambulance dispatches." Inhal Toxicol **24**(12): 858-867.

Viel, J. F., Y. Mallet, C. Raghoumandan, P. Quénel, P. Kadhel, F. Rouget and L. Multigner (2019). "Impact of Saharan dust episodes on preterm births in Guadeloupe (French West Indies)." Occup Environ Med **76**(5): 336-340.

Viel, J. F., L. Michineau, C. Garbin, C. Monfort, P. Kadhel, L. Multigner and F. Rouget (2020). "Impact of saharan dust on severe small for gestational births in the caribbean." American Journal of Tropical Medicine and Hygiene.

Vodonos, A., M. Friger, I. Katra, L. Avnon, H. Krasnov, P. Koutrakis, J. Schwartz, O. Lior and V. Novack (2014). "The impact of desert dust exposures on hospitalizations due to exacerbation of chronic obstructive pulmonary disease." AIR QUALITY ATMOSPHERE AND HEALTH **7**(4): 433-439.

Vodonos, A., M. Friger, I. Katra, H. Krasnov, D. Zahger, J. Schwartz and V. Novack (2015). "Individual Effect Modifiers of Dust Exposure Effect on Cardiovascular Morbidity." PLOS ONE **10**(9).

Wang, C. H., C. S. Chen and C. L. Lin (2014). "The threat of Asian dust storms on asthma patients: a population-based study in Taiwan." Glob Public Health **9**(9): 1040-1052.

Wang, J., S. Li, S. Wang and K. Shang (2015). "Effects of Long-Term Dust Exposure on Human Respiratory System Health in Minqin County, China." Arch Environ Occup Health **70**(4): 225-231.

Wang, Y., R. Y. Wang, J. Ming, G. X. Liu, T. Chen, X. F. Liu, H. X. Liu, Y. H. Zhen and G. D. Cheng (2016). "Effects of dust storm events on weekly clinic visits related to pulmonary tuberculosis disease in Minqin, China." ATMOSPHERIC ENVIRONMENT **127**: 205-212.

Wang, Y. C. and Y. K. Lin (2015). "Mortality associated with particulate concentration and Asian dust storms in Metropolitan Taipei." ATMOSPHERIC ENVIRONMENT **117**: 32-40.

Watanabe, M., T. Igishi, N. Burioka, A. Yamasaki, J. Kurai, H. Takeuchi, T. Sako, A. Yoshida, K. Yoneda, Y. Fukuoka, M. Nakamoto, Y. Hasegawa, H. Chikumi, S. Matsumoto, S. Minato, K. Horasaki and E. Shimizu (2011). "Pollen augments the influence of desert dust on symptoms of adult asthma patients." Allergol Int **60**(4): 517-524.

Watanabe, M., J. Kurai, T. Igishi, A. Yamasaki, N. Burioka, H. Takeuchi, T. Sako, H. Touge, M. Nakamoto, Y. Hasegawa, H. Chikumi, S. Matsumoto, C. Yamasaki, S. Minato, Y. Ueda, K. Horasaki, T. Watanabe and E. Shimizu (2012). "Influence of Asian Desert Dust on Lower Respiratory Tract Symptoms in Patients with Asthma over 4 Years." Yonago Acta Med **55**(2): 41-48.

Watanabe, M., J. Kurai, H. Sano and E. Shimizu (2015). "Effect of exposure to an Asian dust storm on fractional exhaled nitric oxide in adult asthma patients in Western Japan." Journal of Medical Investigation.

Watanabe, M., J. Kurai and E. Shimizu (2014). "Influence of Asian dust storm on asthma in western Japan." Genes and Environment.

Watanabe, M., J. Kurai, K. Tomita, H. Sano, S. Abe, R. Saito, S. Minato, T. Igishi, N. Burioka, T. Sako, K. Yasuda, M. Mikami, S. Kurita, H. Tokuyasu, Y. Ueda, T. Konishi, A. Yamasaki, S. Aiba, M. Oshimura and E. Shimizu (2014). "Effects on asthma and induction of interleukin-8 caused by Asian dust particles collected in western Japan." J Asthma **51**(6): 595-602.

Watanabe, M., H. Noma, J. Kurai, K. Kato and H. Sano (2021). "Association with Ambient Air Pollutants and School Absence Due to Sickness in Schoolchildren: A Case-Crossover Study in a Provincial Town of Japan." Int J Environ Res Public Health **18**(12).

Watanabe, M., H. Noma, J. Kurai, K. Kato, H. Sano, T. Tatsukawa, H. Nakazaki, A. Yamasaki and E. Shimizu (2016). "Association between pulmonary function and daily levels of sand dust particles assessed by light detection and ranging in schoolchildren in western Japan: A panel study." ALLERGOLOGY INTERNATIONAL **65**(1): 56-61.

Watanabe, M., H. Noma, J. Kurai, H. Sano, K. Iwata, D. Hantan, Y. Tohda and E. Shimizu (2017). "Association of Short-Term Exposure to Ambient Fine Particulate Matter with Skin Symptoms in Schoolchildren: A Panel Study in a Rural Area of Western Japan." Int J Environ Res Public Health **14**(3).

Watanabe, M., H. Noma, J. Kurai, H. Sano, M. Mikami, H. Yamamoto, Y. Ueda, H. Touge, Y. Fujii, T. Ikeda, H. Tokuyasu, T. Konishi, A. Yamasaki, T. Igishi and E. Shimizu (2016). "Effect of Asian dust on pulmonary function in adult asthma patients in western Japan: A panel study." Allergol Int **65**(2): 147-152.

Watanabe, M., H. Noma, J. Kurai, H. Sano, R. Saito, S. Abe, Y. Kimura, S. Aiba, M. Oshimura, A. Yamasaki and E. Shimizu (2015). "Decreased pulmonary function in school children in Western Japan after exposures to Asian desert dusts and its association with interleukin-8." Biomed Res Int **2015**: 583293.

Watanabe, M., H. Noma, J. Kurai, H. Sano, Y. Ueda, M. Mikami, H. Yamamoto, H. Tokuyasu, K. Kato, T. Konishi, T. Tatsukawa, E. Shimizu and H. Kitano (2016). "Differences in the effects of Asian dust on pulmonary function between adult patients with asthma and those with asthma-chronic obstructive pulmonary disease overlap syndrome." Int J Chron Obstruct Pulmon Dis **11**: 183-190.

Watanabe, M., H. Noma, J. Kurai, A. Shimizu, H. Sano, K. Kato, M. Mikami, Y. Ueda, T. Tatsukawa, H. Ohga, A. Yamasaki, T. Igishi, H. Kitano and E. Shimizu (2015). "Association of Sand Dust Particles with Pulmonary Function and Respiratory Symptoms in Adult Patients with Asthma in Western Japan Using Light Detection and Ranging: A Panel Study." INTERNATIONAL JOURNAL OF ENVIRONMENTAL RESEARCH AND PUBLIC HEALTH **12**(10): 13038-13052.

Watanabe, M., A. Yamasaki, N. Burioka, J. Kurai, K. Yoneda, A. Yoshida, T. Igishi, Y. Fukuoka, M. Nakamoto, H. Takeuchi, H. Suyama, T. Tatsukawa, H. Chikumi, S. Matsumoto, T. Sako, Y. Hasegawa, R. Okazaki, K. Horasaki and E. Shimizu (2011). "Correlation between Asian dust storms and worsening asthma in Western Japan." Allergol Int **60**(3): 267-275.

Williams, P. L., D. L. Sable, P. Mendez and L. T. Smyth (1979). "SYMPTOMATIC COCCIDIOIDOMYCOSIS FOLLOWING A SEVERE NATURAL DUST STORM - OUTBREAK AT THE NAVAL AIR STATION, LEMOORE, CALIF." CHEST **76**(5): 566-570.

Wong, M. S., H. C. Ho, L. Yang, W. Shi, J. Yang and T. C. Chan (2017). "Spatial variability of excess mortality during prolonged dust events in a high-density city: a time-stratified spatial regression approach." Int J Health Geogr **16**(1): 26.

Woringer, M., N. Martiny, S. Porgho, B. W. Bicaba, A. Bar-Hen and J. E. Mueller (2018). "Atmospheric Dust, Early Cases, and Localized Meningitis Epidemics in the African Meningitis Belt: An Analysis Using High Spatial Resolution Data." ENVIRONMENTAL HEALTH PERSPECTIVES **126**(9).

Yang, C. Y. (2006). "Effects of Asian dust storm events on daily clinical visits for conjunctivitis in Taipei, Taiwan." J Toxicol Environ Health A **69**(18): 1673-1680.

Yang, C. Y., Y. S. Chen, H. F. Chiu and W. B. Goggins (2005). "Effects of Asian dust storm events on daily stroke admissions in Taipei, Taiwan." Environ Res **99**(1): 79-84.

Yang, C. Y., M. H. Cheng and C. C. Chen (2009). "Effects of Asian dust storm events on hospital admissions for congestive heart failure in Taipei, Taiwan." J Toxicol Environ Health A **72**(5): 324-328.

Yang, C. Y., S. S. Tsai, C. C. Chang and S. C. Ho (2005). "Effects of Asian dust storm events on daily admissions for asthma in Taipei, Taiwan." Inhal Toxicol **17**(14): 817-821.

Yitshak-Sade, M., V. Novack, I. Katra, R. Gorodischer, A. Tal and L. Novack (2015). "Non-anthropogenic dust exposure and asthma medication purchase in children." EUROPEAN RESPIRATORY JOURNAL **45**(3): 652-660.

Yoo, Y., J. T. Choung, J. Yu, D. K. Kim and Y. Y. Koh (2008). "Acute effects of Asian dust events on respiratory symptoms and peak expiratory flow in children with mild asthma." J Korean Med Sci **23**(1): 66-71.

Yu, H. L., L. C. Chien and C. H. Yang (2012). "Asian dust storm elevates children's respiratory health risks: a spatiotemporal analysis of children's clinic visits across Taipei (Taiwan)." PLoS One **7**(7): e41317.

Yu, H. L., C. H. Yang and L. C. Chien (2013). "Spatial vulnerability under extreme events: a case of Asian dust storm's effects on children's respiratory health." Environ Int **54**: 35-44.

Zauli Sajani, S., R. Miglio, P. Bonasoni, P. Cristofanelli, A. Marinoni, C. Sartini, C. A. Goldoni, G. De Girolamo and P. Lauriola (2011). "Saharan dust and daily mortality in Emilia-Romagna (Italy)." Occup Environ Med **68**(6): 446-451.
